# Slip rates along the narrow Magallanes Fault System, Tierra Del Fuego Region, Patagonia

**DOI:** 10.1038/s41598-020-64750-6

**Published:** 2020-05-18

**Authors:** Francisca B. Sandoval, Gregory P. De Pascale

**Affiliations:** Departamento de Geología, Facultad de Ciencias Físicas y Matemáticas (FCFM), Universidad de Chile, Plaza Ercilla 803, Santiago, RM Chile

**Keywords:** Natural hazards, Structural geology, Geomorphology, Seismology, Tectonics

## Abstract

The up to 1000 km-long Magallanes Fault System (MFS) is the southernmost onshore strike-slip plate boundary and located between the South American and Scotia Plates. Slip-rates, a key factor for understanding neotectonics and seismic hazard are only available there from geodetic models. In this study, we present the first direct geologic evidence of MFS slip rates. Late-Cenozoic slip rates along the main MF is 5.4 ± 3.3 mm/yr based on lithologic geological separations found in regional mapping. Late-Quaternary deformation from offset geomorphologic markers was documented along the MFS in Chile and Argentina based on a combination of satellite mapping, fieldwork, and Structure from Motion (SfM) models developed from drone photography. By combining displacements observed in SfM models with regional Late-Quaternary dating, sinistral slip rates are 10.5 ± 1.5 mm/yr (Chile) and 7.8 ± 1.3 mm/yr (Argentina). By comparing our results with regional models, contemporary plate boundary deformation is narrow, approximately ~20–50 km wide from Tierra Del Fuego (TdF) and east (one of the narrowest on Earth), which widens and becoming more diffuse from Cabo Froward north and west (>100 km wide). In addition to the tectonic implications, these faults should be considered important sources of fault rupture and seismic hazard.

## Introduction

**Figure 1:**
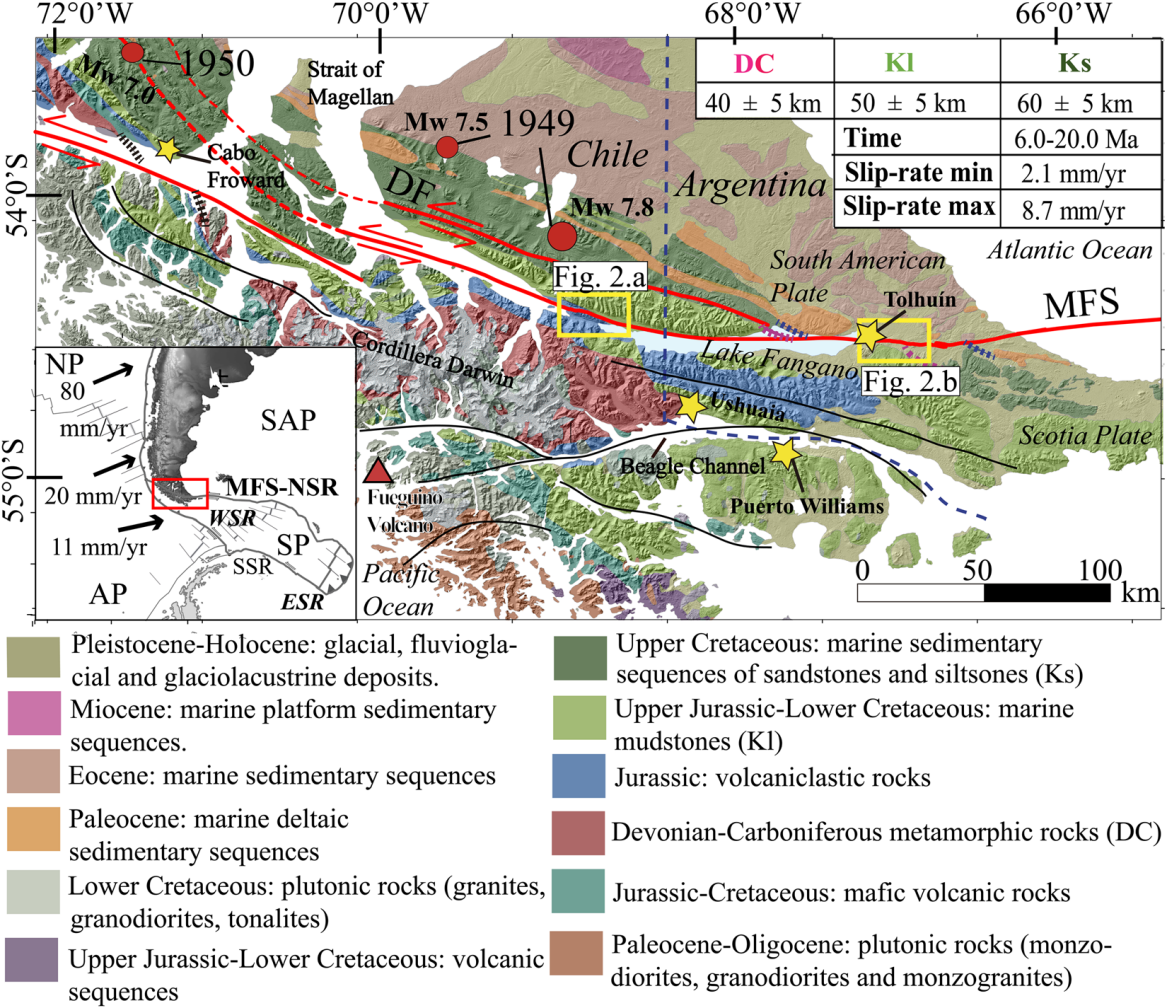
Geological map of the Tierra del Fuego region of Southern Patagonia in Chile and Argentina^5,44,52^. Table indicates Late-Cenozoic MFS slip-rates based on mean values of the plausible sinistral separation of the Devonian-Carboniferous metamorphic unit (DC, red), Upper Jurassic-Lower Cretaceous marine sequences unit (Kl, light green) and Upper Cretaceous marine sedimentary sequences (Ks, dark green) and proposed ages for the beginning of activity (20.0 to 6.0 Ma)^17^. Dotted lines are the projection of the contacts to the MFS. Yellow boxes are field study areas. Red circles indicate historical epicenters^27^. Blue dotted line indicates the international border. Black solid lines indicate lineaments possibly related to tectonic activity^6^. Inset map shows the tectonic configuration between Southern South America (i.e. Patagonia) and the Antarctic Peninsula. MF: Magallanes-Fagnano Fault, DFS: Deseado Fault, NP: Nazca Plate, SAP: South American Plate, AP: Antarctic Plate, SP: Scotia Plate, NSR: North Scotia Ridge, ESR: East Scotia Ridge, SSR: South Scotia Ridge, WSR: West Scotia Ridge. Plate velocities shown with arrows in mm/yr^15,24^. Base hillshade was generated with ESRI ArcMap v.10.3 software (under fair terms of use, https://www.esri.com/en-us/legal/copyright-trademarks)^58^ using a digital elevation model downloaded from ALOSPALSAR Global Radar Imagery with 12.5 m resolution (https://asf.alaska.edu/data-sets/sar-data-sets/alos-palsar/)^55^.

Plate boundary faults are first-order neotectonic structures which accommodate large proportions of crustal scale deformation along narrow zones and are important sources of seismic hazard. At the southern end of South American, the Patagonian Andes is characterized by the curvature of the Fuegian belt, which bends from an N-S trending structural trend (at ~53°15′S) to a more W-E strike along the Island of Tierra Del Fuego^1,2^ (TdF; Fig. 1). There, a continental-scale system of transform faults^3^ called the either the Magallanes-Fagnano Fault System or Magallanes Fault System^4–6^ (MFS), accommodates sinistral (left-lateral) motion along the plate boundary between the Scotia and South American Plates^5–7^. Historical large magnitude shallow (<15 km) crustal earthquakes (Fig. 1) along the MFS in 1879 (Mw 7.0–7.5), 1949 (Ms 7.8 and Ms 7.5), 1950 (Mw 7.0) and 1970 (Mw 7.2)^8,9^ (Fig. 1) demonstrates the seismic hazard from the MFS. Limited paleoseismic records estimate recurrence intervals for high magnitude earthquakes from 750 to 2,000 years^10–12^. To date, faults within the MFS are poorly mapped or concealed due to being submarine or found within dense forest (except further to the east of TdF), with slip rate estimates only derived from modeling. Slip rate estimations from geodetic data are based on a limited number of GPS stations over limited timeframes (Fig. 1; 6.6 ± 1.3 mm/yr^13^; 5.9 ± 0.2 mm/yr^14^; 9.6 ± 1.4 mm/yr^15^). This demonstrates the need for geological data to better understand long-term neotectonics along this plate boundary and evaluate the geophysical models. This paper presents new field and remote sensing mapping and offset data that provide geological and geomorphic slip rates along this plate boundary.

The MFS is a up to 1000 km long transform boundary^5,7^ with limited neotectonic data. The absence of data is likely because much of the fault system is submarine or below lakes, only ~65 km of the MFS are onshore in Chile and ~60 km in Argentina respectively, or hidden within dense forest (Fig. 1). Broad east-west trending fault segments were identified because of their geomorphological expression^6^. West of TdF, the main fault trace passes through towards the Almirantazgo Fiord until it intersects the Chile Trench (52°S, 76°W), 500 km from TdF, defining the triple junction between the South American, Antarctic and Nazca Plates^4,6^, while the plate boundary east of TdF the extends along the North Scotia Ridge (NSR) up to 1800 km (Fig. 1).

The relative motion history between South America and the Antarctic Peninsula could have commenced in the Upper Cretaceous and the associated tectonic motions likely triggered the development of the Patagonian bend^16^. Other authors suggest that the development of the current plate boundary, the MFS-NSR, occurred later due to the onset of seafloor spreading in the western Scotia Sea and the opening of Drake Passage in the Eocene-Oligocene^4^. According to this model, the strike-slip deformation in TdF started after 30 Ma, superimposing the contractional deformation dominant during the Cretaceous. On the other hand, AMS dataset shows that from at least the Early Cretaceous until the end of the Oligocene the tectonic regime at the Southern Andes yielded continuous contraction, implying that the uplift and exhumation of the Cordillera Darwin occurred within a compressive regional tectonic regime, rather than in a strike-slip setting^2^. Between 46 to 20 Ma, the spreading rates in the Western Scotia ridge increased, changed to an WNW-ESE direction, resulting in a transpression along the Northern Scotia Ridge (NSR)^17^.^.^ At 20 Ma, a change in the motion to an W-E direction lead to the initiation of spreading at the East Scotia Ridge, and thus the onset of MFS and an important drop in the spreading rates on the West Scotia Ridge^17,18^. However, MFS-NSR transform system development may have initiated at ~6 Ma^19^, coeval with cessation of western Scotia seafloor spreading and a significant increase in the Eastern Scotia ridge activity^17,18^ (Fig. 1), which led to the current plate geometry with the inactive West Scotia Ridge and ongoing sinistral motion.

Individual faults within the MFS in TdF around Lake Fagnano (which is also called Lake Cami in Chile) are mapped and defined based on seismic profiles interpretation and bathymetric data and regional mapping. The main structures are the master Magallanes fault (MF), Deasado fault (DF), Hope-Catamarca-Knokeke fault (HF), Río Turbio-Las Pinturas fault, and San Rafael fault^5,6,10,20,21^. Broadly W-E trending fault segments were identified mostly by remote sensing and geomorphic expression along the valleys of surrounding Lake Fagnano both in Chile^5,19–21^ and Argentina^10,22^. Nevertheless, the exact location of the onshore faults in some areas is still poorly known, especially where tectonic lineaments are hidden by recent deposits including peat bogs and extensive disturbance from introduced beavers^10,22^. West of TdF, according to fault slip data within the Magallanes fold and thrust belt near the Strait of Magellan (Fig. 1), strike slip and oblique-slip normal faults sets reflects a regional bulk transtension with localized mechanical anisotropies defined by the structural trend of the orogen^19^.

**Figure 2:**
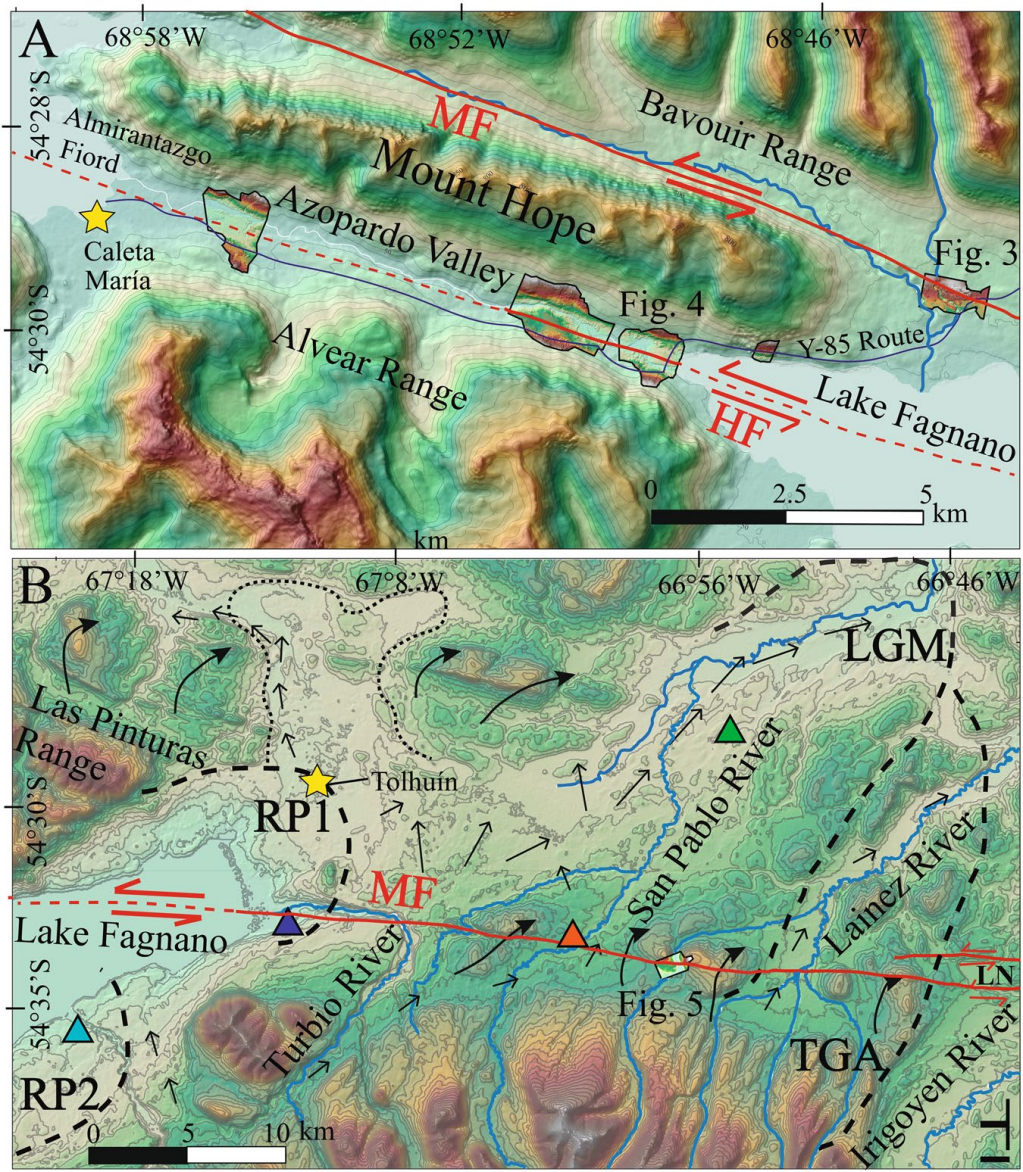
(**a**) Chilean study area in TdF. SfM DEMs created from drone photography collected during fieldwork shown over the 12.5 m resolution regional DEM. MF: Magallanes fault, HF: Hope fault. Solid red lines are observed faults and dashed red lines are inferred faults. (**b**) Argentina study area in TdF. Thick dashed black lines show glacial boundaries while thin dotted lines show ice disintegration landscape area triggered by the ice retreat^32^. MF: Magallanes Fault, LGM: Last Glacial Maximum; TGA: Tributary glaciers area, RP1: Recessional Phase 1, RP2: Recessional Phase 2. LN: Laguna Negra Bog. Thin black arrows indicate flow direction of outwash paleo-streams triggered by the melting of Fagnano Paleo-lobe post RP1^32^. Thick black arrows indicate glacial overridden hills^32^. Colored triangles are radiocarbon dating of peat bogs (green, 13.8-14.4 ka; orange, 13.4-13.9 ka; light green, 13.8-14.2 ka; blue, 8.9-9.3 ka)^32^. 50 m contours shown. Base hillshade was generated with ESRI ArcMap v.10.3 software (under fair terms of use, https://www.esri.com/en-us/legal/copyright-trademarks)^58^ using a digital elevation model downloaded from ALOSPALSAR Global Radar Imagery with 12.5 m resolution (https://asf.alaska.edu/data-sets/sar-data-sets/alos-palsar/)^55^. SfM models were generated using the Agisoft Standard Photoscan Pro 1.3.2 (2018) (https://www.agisoft.com)^56^.

Fault mapping of the onshore MFS was reported by previous authors based on kinematic data, bathymetric mapping and seismic profiling interpretation and geological offsets both in Chile and Argentina^5,19–21^. Klepeis^5^ carried out the first MFS mapping in Chile between the eastern portion of Lake Fagnano and the Almirantazgo Fiord encompassing the Mount Hope area (Figs. 1 and 2). Three MFS sinistral segments offset the thrust contact between Upper Jurassic volcaniclastic rocks of the Lemaire Fm. and Upper Jurassic to Lower Cretaceous tuffs over 20-25 km. One clear geomorphic expression of the MF is in the area around Lake Fagnano is the disrupted lakebed stratigraphy in the center of the lake^20^. However other faults are proposed in the area. The San Rafael fault occurs along the southwestern shore of the lake north of Sierra Valdivieso is steeply dipping^20^. Las Pinturas fault, like the San Rafael fault, trends parallel to the lake shore forming a releasing step-over and connects with the Rio Turbio fault east of Lake Fagnano, and both are sub-vertical fault plane striking W-E. Subordinately, minor sub-parallel lineaments were recognized in the eastern portion of Tierra del Fuego in Argentina^22^, and are related with the master MF with a ~10 km wide shear zone. West of TdF, left-lateral fault segment named Bahía del Indio^19^, which is perhaps the western continuation of the DF, strikes NW of the Strait of Magellan and north of Cabo Froward (Fig. 1). There, a left-lateral left-stepping releasing zone along MFS main fault trace along the Strait of Magellan is proposed^19^. Importantly, fault rocks such as gouge or breccia were not reported from any of these structures.

Moderate to low seismicity^23^ primary due to the Antarctic-Scotia Plate convergence (11 mm/yr) and along the crustal MFS Plate boundary is found here^24^. The instrumental seismicity (from 1999 to present) use only limited permanent seismic stations^25^. Nevertheless, hypocenters of the events related to the MFS are shallow (5 to 10 km), with ~15 km locking depths^13^. The earliest report of historic seismicity was on 2 February 1879, with an intensity of VIII in Ushuaia and estimated magnitude of M 7.0 to 7.5^8^. A prehistoric event before European colonization exists according to an Ona legend (a.k.a. Yaghán), which provides important indigenous traditional knowledge about neotectonics in the region^26^. The largest recorded event from global seismic networks was the December 17, 1949 Ms 7.8 earthquake^8,24^ with a Ms 7.5 foreshock^8,24,27^ (Fig. 1). Coseismic effects from 1949 were extensive. Landslides occurred along the west coast of TdF^5,8^. Drowned forest (liquefaction and lateral spread?) were submerged along the eastern side of Lake Fagnano^10,26^, while scarps and tsunamis were reported along the Strait of Magellan^27^. 1949 fault rupture evidence are proposed in the Quaternary deposits east of Lake Fagnano^10^. Post-1949, in 1970 a Ms 7.0 earthquake occurred along the eastern NSR with left-lateral slip on a sub-vertical W-E plane^24^ (Fig. 1).

Paleoseismic data here is limited but provides basic insight into MFS behaviour^26^, using dendrochronology in Lenga trees (N*othofagus pumilio*) along MF surfaces ruptures in Argentina near Lake Fagnano which report abrupt changes in tree rings (i.e. from concentric to asymmetric rings) in 1883 ± 5 and 1941 ± 10. These results coincide with the 1879 and 1949 TdF earthquakes, indicating that the MF was the source of this events. A MF secondary fault trace was trenched^10^, with at least 3 paleo-events documented over 9 ka. High-resolution seismic profiles and cores to date slope failures and mega-turbidities in Lake Fagnano identified submarine ruptures and at least 19 submarine landslides in the last 12 ka^11^. Shallow geophysics and dating of faults in two strand-plains north of the Strait of Magellan, reported ages of 0.9, 1.3, 2.4, 3.0, 3.9 and 6.4 ka BP and a recurrence rate of 0.9 ka^12^. Along the main MF, left-lateral displacements of 0.4 m up to 6 m were reported according to observations from locals living there in 1949^10,13^. East of Tolhuín, a 5 to 11 m high a multi-event scarp exposes successive terraces of Quaternary glacial-fluvial materials^10^ with coaxial grabens, tension gashes with *en échelon* geometry and mole tracks^9^.

Although geologic rates across the MFS are not available, GPS measurements allow model-derived rates, with most GPS data with limited spatial resolution and limited recorded periods^13,14^ (Supplemental Material. A network of GPS stations in TdF provided a modelled slip-rate of 6.6 ± 1 to 6.8 ± 1 mm/yr for the MFS based on a set of 20 GPS stations^13,28^. These results and strain analysis suggest that main crustal deformation zone in TdF is concentrated along the MFS in a zone which is ~50 km-wide^14^. The MORVEL plate boundary displacement rates model along the NSR gives rates that decrease from 9.6 ± 1.4 mm/yr (1 *σ*) in the west (65°W) to 8.9 ± 1.2 mm/yr in the east along the MFS (35°W)^15^.

At least five major glaciations were identified in TdF region since the Pleistocene, based on the glacial deposits found located along the Strait of Magellan (1 Ma to 23 ka^29–31^). Around Lake Fagnano the ice network in this region developed due mostly due to Fueguian Andes topography^31–33^. The main ice bodies were outlet glaciers flowing eastwards from the Darwin Cordillera through different lobes (i.e. Magellan, Bahía Inútil, Beagle and Fagnano)^29,32^. During retreat they left an extensive cover of glacial, glacial-fluvial, and glacio-lacustrine deposits^32^. The easternmost glacial termini correspond to the Last Glacial Maximum (LGM) maximum extension in Argentina at 66°45’W along the San Pablo, Ewan and Fuego Valleys^32^. A maximum age for LGM ice advance in TdF was 53.2–43.4 and 37.0–35.2 ka cal B.P^32^ with retreat around 25.7 ka based on the lateral-frontal Río Fuego Valley LGM moraine^32^. In TdF the LGM maximum extension corresponds with recessional stages (1-3)^32^ at ~18 ka and 10.2 ka uncalibrated C^14^ years according to the Bahía Inútil and Strait of Magellan model^30^. The earliest Late Glacial or Recessional Phase 1 (i.e. retreat from LGM), is found at the Tolhuín frontal moraine, east of Lake Fagnano during the first re-advance similar to the Bahía Inútil lobe^30–32^, with an extensive proglacial glacio-fluvial or outwash plain formed by melting of the Fagnano glacier^32^. Radiocarbon ages from the base of a peat bog^32^ would not indicate the minimum absolute age of the ice recession (Río Turbio and Lago Fagnano peat with 9.0 to 9.3 ka and 9.9 to 10.2 ka cal. years B.P. respectively), because debris-covered ice delayed the peat formation by perhaps several thousand years. However, by comparing this major advance or standstill with the “Stage C” in the Bahía Inútil model^30^, the ice would have retreated before 21.7 to 20.4 ka cal B.P. The deposits from Stage C include the glaciofluvial outwash deposits found in the San Pablo, Lainez and Irigoyen Valleys^32^.

Post LGM advances or 2^nd^ Recessional phase was recognized in Lake Fagnano by bathymetric, seismic and coring studies^34^ (Supplemental Material). Ages indicate that the area was free of ice between 14.8 and 12.7 ka cal B.P.^32^, and is comparable with the “Stage D” of the Bahía Inútil model, (pre-17.5 to 16.6 ka cal B.P)^30^. The 3^rth^ recessional phase also known as the Latest Late Glacial, is represented by the Martínez and Chilena moraines located in the western portion of the study area and is the post-LGM stage before ice retreated to the high mountains of Darwin Cordillera^30^. Till here are found up to 700 m a.s.l. on the slopes of tributary valleys^30^. Although no absolutes ages exist for these deposits, from regional mapping they are likely Younger Dryas based on maximum ages of 12.5 to 11.7 ka which aligns with the “Stage E” in the Inútil Bay model^30^. West of TdF, marine data from Almirantazgo Sound indicates deglaciation occurred prior to 14.3 ka cal BP, and became a predominantly saline fiord environment by 9.8 ka cal B.P^33^. Post-glacial retreat, landslides, river deposition and erosion, and more recently the influence of beaver dams control the non-tectonic geomorphic elements found in the modern landscape.

## Results

Geological horizontal separations between lithologic units we identified along the MFS main trace (Fig. 1) suggest that total left-lateral MF slip ranges from 40 ± 5 km to 60 ± 5 km. Previously reported separations are 48 ± 20 km (contact between Ks and Pl)^22^, 25 km (cumulative separation of the contact between Js and Kl^5^) and 80 km (apparent offset in the Patagonian Batholith^4^) (Fig. 1). By combining the slip with age of the rocks, long-term (Late Cenozoic, based on the ages of the units) geological strike-slip slip rates range from 5.4 ± 3.3 mm/yr (2.1 to 8.7 mm/yr). The large range is based on uncertainties in timing from 20.0 Ma to 6.0 Ma for the onset of the MFS sinistral motion^17,18^ (Fig. 1).

**Figure 3:**
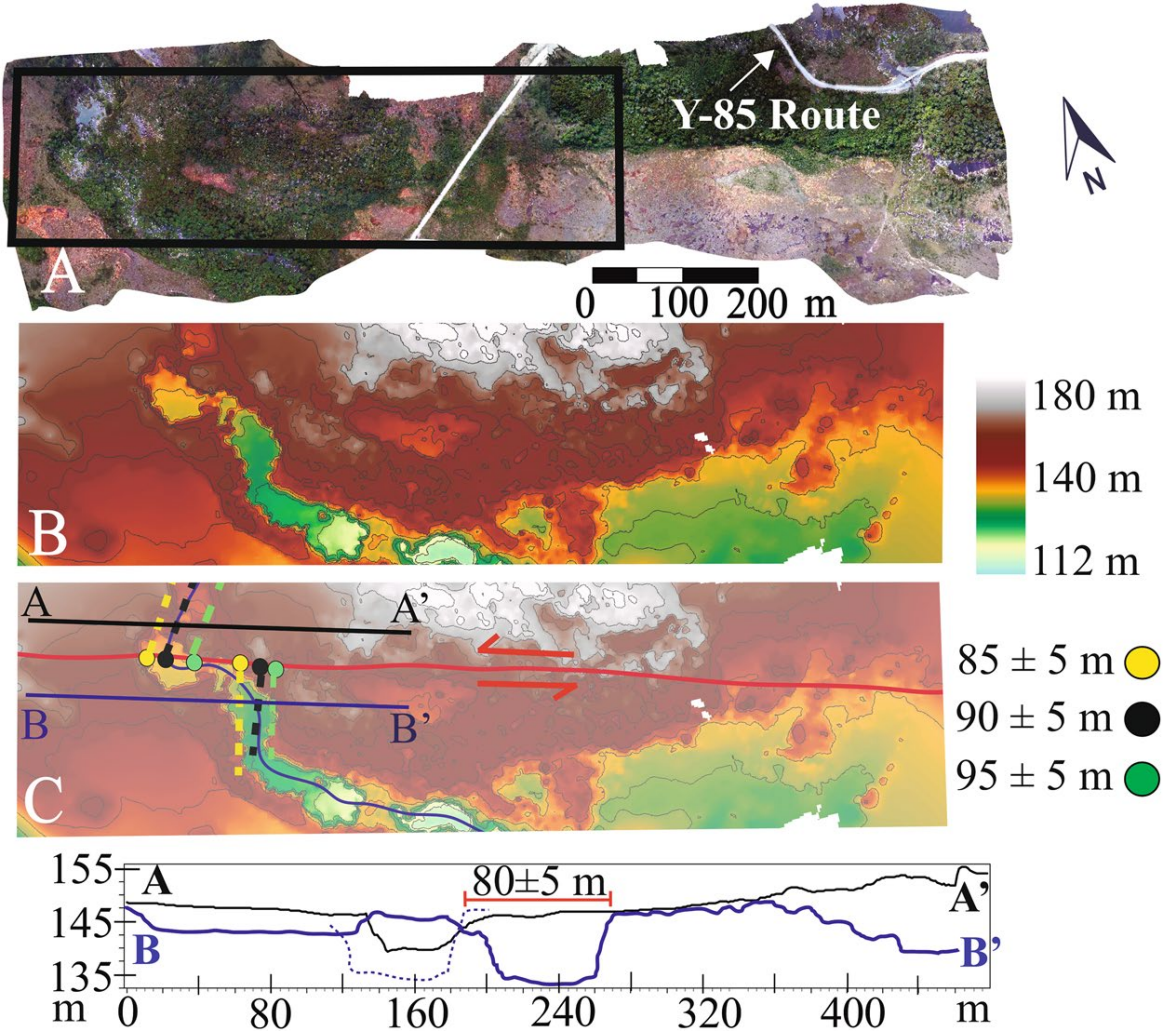
(**a**) SfM Orthophoto along the main trace of the Magallanes Fault near Mont Hope in Chile (Fig. 2a). A 5-7 meter high fault scarp is observed east the road with peat bog deposits and vegetation is aligned along the fault trace. Black box in (a) are the areas shown in detail in B and C. (**b**) High resolution SfM DEM for MF section without interpretation and (**c**) with interpretations and locations of topographic profiles. The topographic profiles (A-A’ in black and B-B’ in blue) parallel to the fault shows the horizontal distance needed for back-slip the channel and glacial deposits north and south the fault trace. The dotted blue line represents the channel in B-B’ after back-slipping (i.e. retro-deformation) to be aligned with the channel in A-A’. The displacements corresponds to the offsets measured in the western (yellow dashed) and eastern (green dashed) limits of the small valley and in the thalweg (black dashed). 5 m contour interval. SfM models were generated using the Agisoft Standard Photoscan Pro 1.3.2 (2018) (https://www.agisoft.com)^56^. Topographic profiles were generated with ESRI ArcMap v.10.3 software (under fair terms of use, https://www.esri.com/en-us/legal/copyright-trademarks)^58^.

The master MF in Chile (Figs. 1 and 2) is found along the north-western shore of Lake Fagnano and is north of Mt. Hope. Here, the trace of the MF is a well-defined-single rectilinear ~70° striking strand with clear evidence of Late Quaternary surface ruptures with a south-facing 5-10 m high scarp with clear vegetation lineaments (Fig. 3). SfM data allowed us to see the channel of a creek along this trace sinistrally offset by the main fault by 80 ± 5 m to 95 ± 5 m (Fig. 3). Offsets were measured in each side of the river and in the thalweg of the channel. Additionally, parallel topographic profiles were made parallel to the fault trace and back-slipped (Fig. 3) to determine best fit. Vertical motions do not appear to be present along the MF here, with the “vertical” scarp being more due to translation of topography which provides the suggestion of a vertical motion. Because there are no major vertical offsets along the channel margins on either side of the MF, it suggests that the MF here is dominantly strike slip (Fig. 3). Elongated strips of undifferentiated till along the northern and southern shores of Lake Fagnano in Chile are cut by the fault and suggest they are the last depositional stage of the Fagnano paleo-glacier before its definitive recession (i.e. Third Recessional phase) once it reached the Azopardo Valley^11,32^. The deposits are up to 12.5 ka, and are correlated with the Younger Dryas in TdF^11^. Because 10.2 ka is proposed as the definitive age of deglaciation for Southern Patagonia it therefore forms a minimum age for these landforms^29^. Thus the deglaciation ages when combined with the displacements (Fig. 3) allow us to determine a long-term (Late Quaternary) geological slip-rate from 7.8 ± 1.1 mm/yr (6.7 to 8.9 mm/yr) along the MF in Chile.

**Figure 4:**
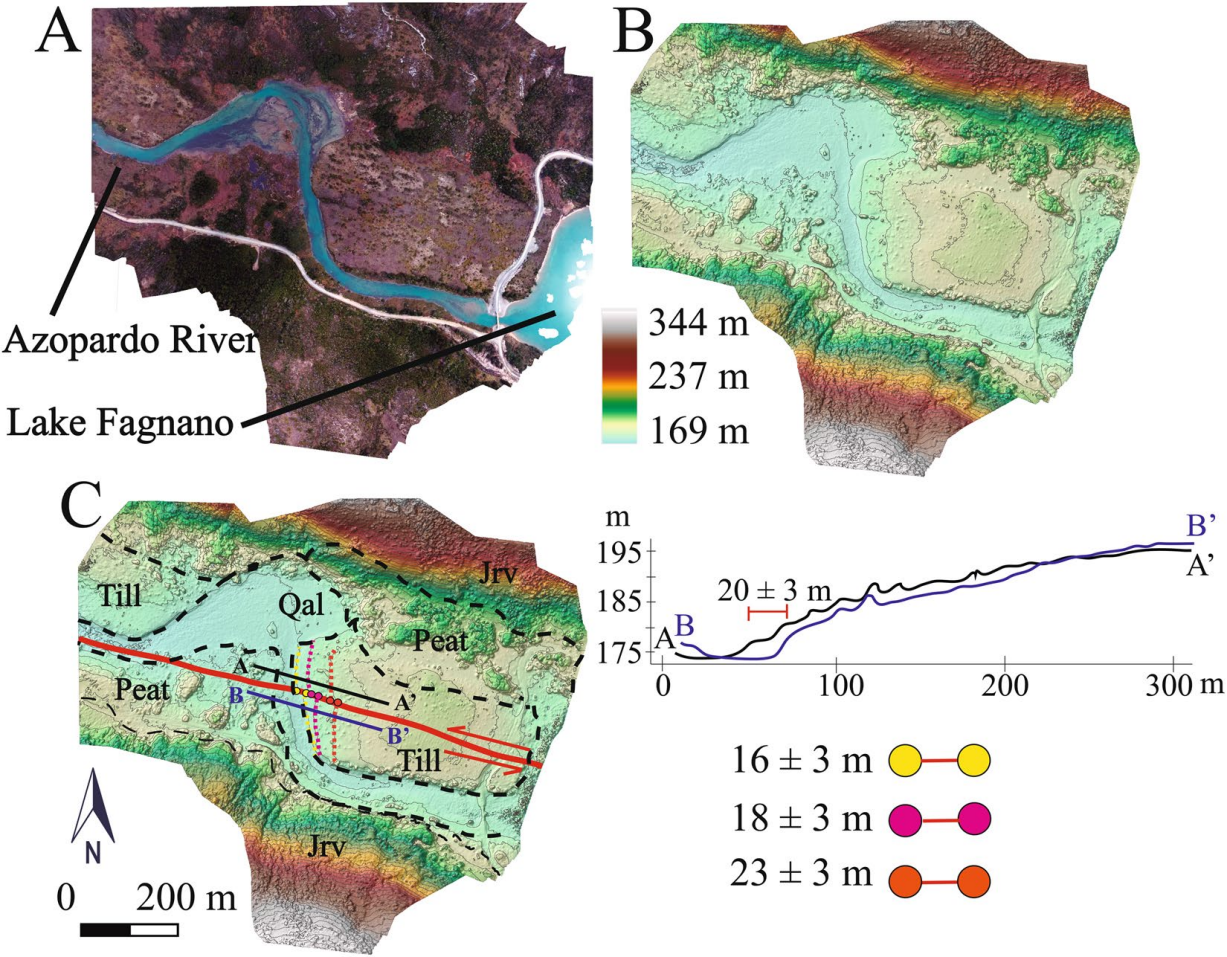
(**a**) Orthophoto derived from SfM data along the Hope fault (HF) at the western edge of Lake Fangano where the Azopardo River drains the lake in Chile to the Straight of Magellan in the west. Note the scarp cutting across the surface parallel with the river flow and displaced terrace risers. Qal: Quaternary alluvial deposits, Till: undifferentiated till, Jrv: Jurassic volcaniclastic rocks from Lemaire fm. (**b**) High resolution SfM DEM for HF section without interpretation with 5 m contours intervals. (**c**) Interpretations on the DEM showing displaced river terraces (in pink, yellow, and orange). Solid red lines indicate the HF fault trace observed along the valley. Topographic profiles (A-A’ in black and B-B’ in blue) parallel to the HF. Topographic profile shows the sinistral displacement of the terraces along the HF. SfM models were generated using the Agisoft Standard Photoscan Pro 1.3.2 (2018) (https://www.agisoft.com)^56^. DEM was processed with ESRI ArcMap v.10.3 software (under fair terms of use, https://www.esri.com/en-us/legal/copyright-trademarks)^58^.

The Hope Fault (HF^5,20^), located only ~5 km south and sub-parallel with the MF was evaluated in the Azopardo Valley south of Mount Hope (Figs. 2 and 4). Onshore it is ~11 km long but likely continues offshore within the Almirantazgo Fiord according to seismic reflection data from the Strait of Magellan^4^, and to the east trending parallel or merging with the MF in Lake Fagnano. SfM data allowed identification of a clear fault trace (although less prominent than the nearby MF) along the centre of the valley (parallel with the river) including two displaced river terrace risers in till with offsets from 16 ± 3 m to 23 ± 2 m in the central-eastern portion of the valley (Fig. 4). The topographic profiles derived from the DEM data show these sinistral displacements clearly (Fig. 4). When combined with the deglaciation records outlined above for the MF in Chile, these data suggest Late-Pleistocene HF slip-rates from 1.7 ± 0.4 mm/yr (1.3 to 2.1 mm/yr).

**Figure 5:**
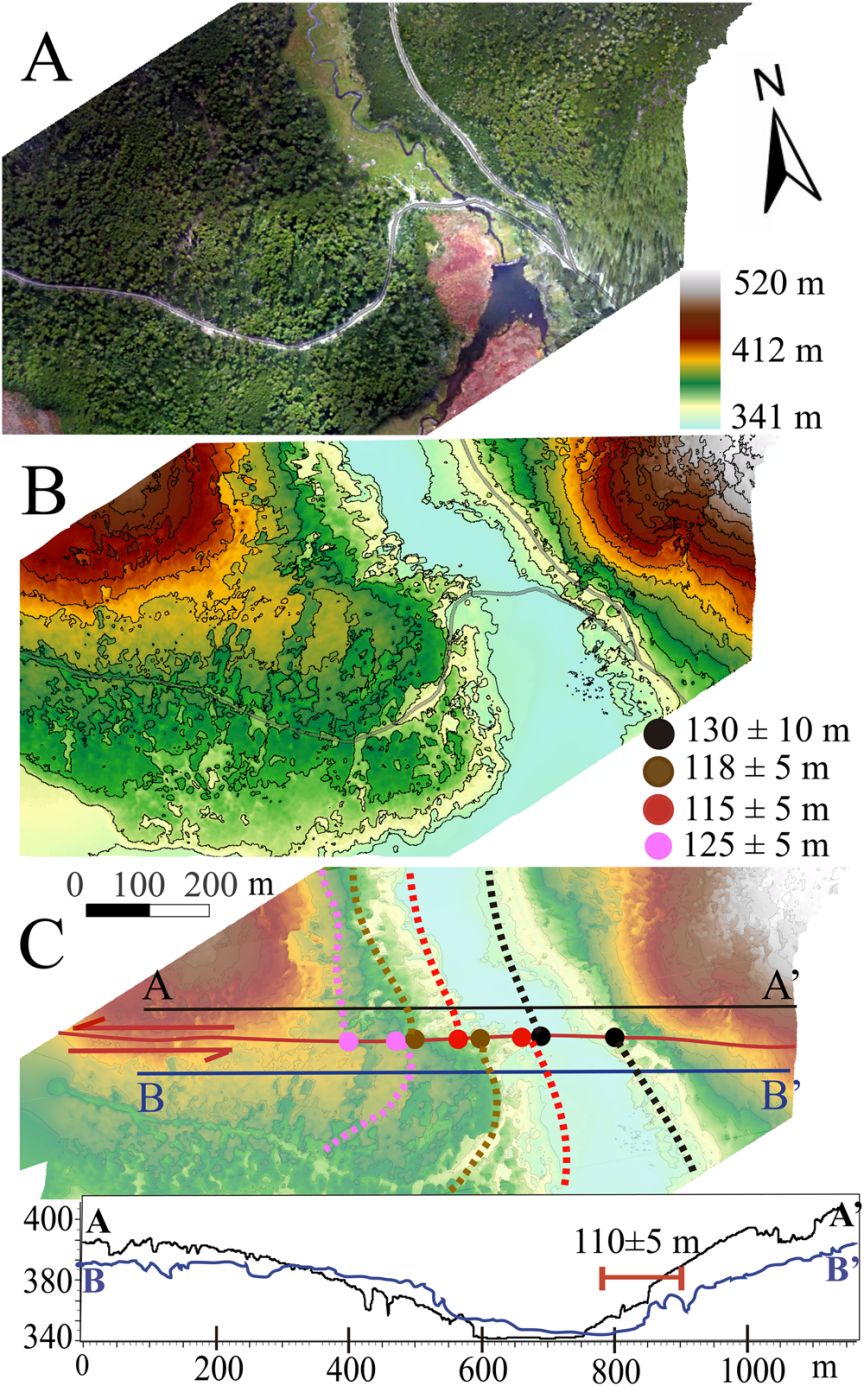
(**a**) Orthophoto derived from SfM data along the master Magallanes Fault (MF) at the Lainez River in Argentina where the river cuts glacial and recent river deposits, (**b**) High resolution SfM DEM for MF section without interpretation and 5 m contour intervals. (**c**) Interpretations on the DEM showing the left-lateral displaced channel, the offsets observed both in recent (modern) river deposits and fluvio-glacial deposits. The topographic profiles (A-A’ in black and B-B’ in blue) show the sinistral motion along the MF and dotted line shows horizontal distance needed for back-slip the channel north and south the fault trace. The displacements corresponds to the offsets measured in the 350 m (red and black), 380 m (brown) and 400 m (pink) contours. SfM models were generated using the Agisoft Standard Photoscan Pro 1.3.2 (2018) (https://www.agisoft.com)^56^. DEM was processed with ESRI ArcMap v.10.3 software (under fair terms of use, https://www.esri.com/en-us/legal/copyright-trademarks)^58^.

**Figure 6:**
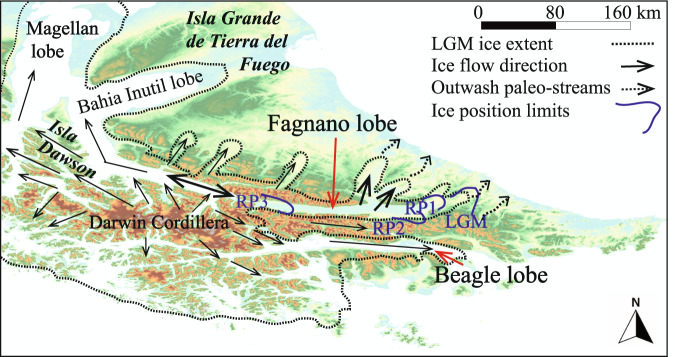
A compilation map (with Lake Fagano in the center of the Fagnano lobe) developed for this work to document Late-Quaternary glaciations of Southern Patagonia that was used to constrain timing for total slip observed in offsets found during fieldwork and shown in Figs. 2–5. Base map of South America from ASTER G-DEM 30 m/pixel resolution. Dashed black lines are LGM maximum extent in Southern Patagonia (with retreat at ~25.7 ka)^29^, whereas dark blue lines indicated the maximum extension for the ice stillstands and retreat (LGM maximum extension and recessional phases)^32^. LGM: Last Glacial Maximum, RP1: First Recessional Phase or earliest Late glacial (i.e. Tolhuin Recessional Phase); RP2: Second Recessional Phase or Late Glacial (17.5 ka – 16.6 ka); RP3: Third Recessional Phase or Latest Late Glacial (which may perhaps be related to Younger Dryas) and occurred around 12.5 to 11.7 ka^29,32^. Base hillshade was generated with ESRI ArcMap v.10.3 software (under fair terms of use, https://www.esri.com/en-us/legal/copyright-trademarks)^58^ using a digital elevation model downloaded from ALOSPALSAR Global Radar Imagery with 12.5 m resolution (https://asf.alaska.edu/data-sets/sar-data-sets/alos-palsar/)^55^.

East of Lake Fagnano in Argentina, the MF extends east to the Claro and Lainez Rivers^10^ (Fig. 2b). The eastern tip of the trace reaches the Laguna Negra^21,22^ bog area (Fig. 2), where forms part of the southern boundary of an inferred 13-km-long pull-apart basin (based on two sub-parallel strands of the main MF) with an overlapping left-stepping extensional geometry. Despite the thick vegetation, the main MF scarp is visible in satellite images as a clear, straight, roughly west to east linear geomorphic trace. In the SfM derived DEMs obtained where the western branch of the Lainez River crosses the fault (Figs. 2 and 5), the MF strikes 80° with clear left-lateral offsets of the River channel and adjacent hillslopes based on retro-deformation (i.e. backslip) of the SfM models (Fig. 5). Geomorphological offsets from 110 ± 5 m to 130 ± 10 m (Fig. 5) were measured from SfM model data along channel margins considering possible post-events modification by sedimentary process. Fieldwork reveals classic strike slip tectonic geomorphology with sag ponds, and shutter ridges, in addition to clear sinistral offsets (Supplemental Material). The MF here cuts LGM glacial and post-glacial deposits related to Glacial Recessional phase 1^32^ superimposed in some segments by recent alluvial deposits (Fig. 2). A maximum age for these deposits is the LGM deglaciation, which began shortly after 17.8 ka^30^ and a minimum age of 13.4 ka based on radiocarbon dating of basal peat bogs^32^ (Figs. 2 and 6), which started growing only after the area was ice-free. The offsets measured here combined with the available regional age data (Fig. 6) provide a MF Late-Quaternary slip rate of 7.8 ± 1.3.

### Discussion regarding the fast slipping and narrow MFS plate boundary

**Figure 7:**
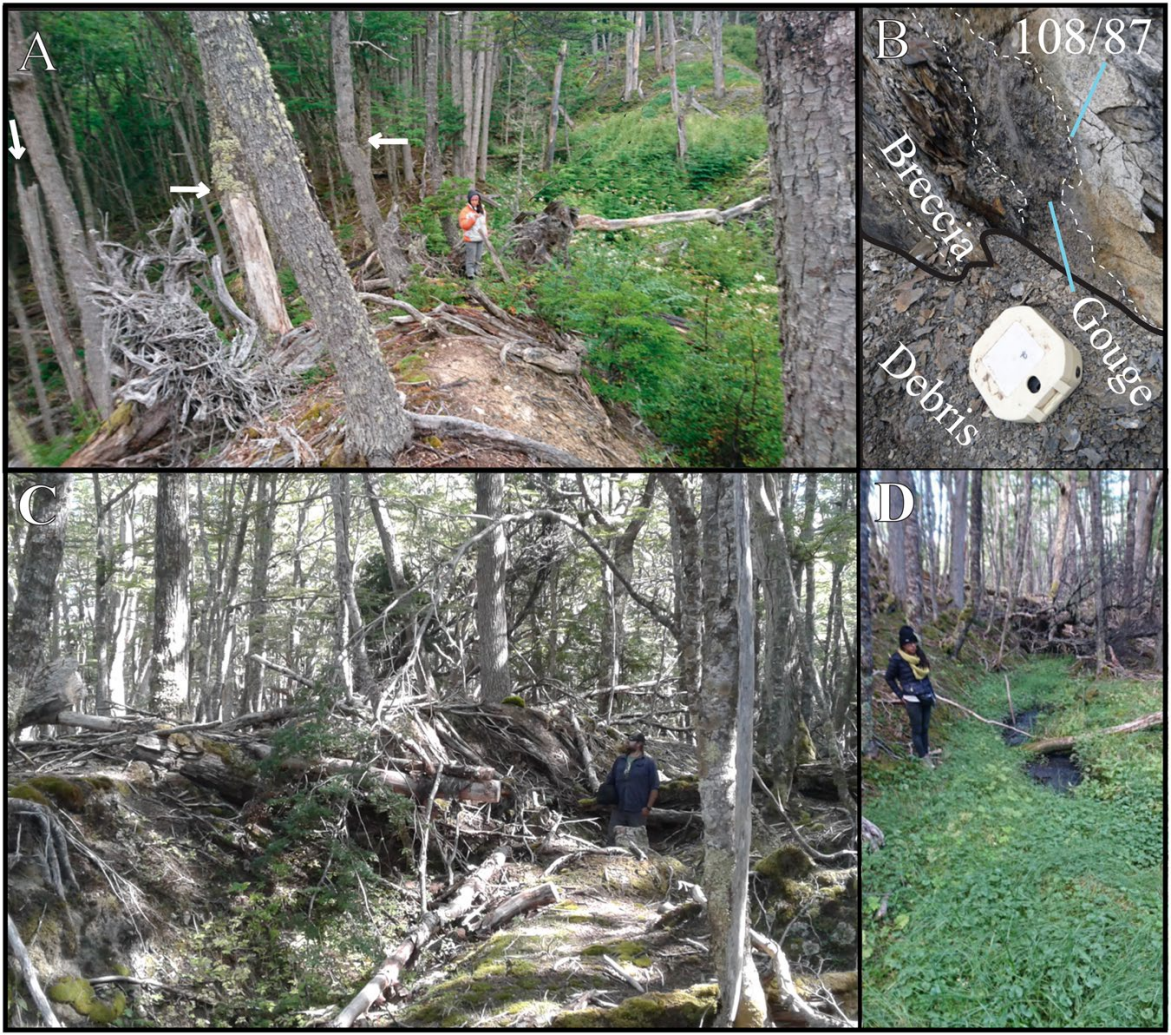
Field photos documenting tectonic geomorphology along faults within the MFS. (**a**) Photograph looking along an shutter ridge (and uphill facing scarp) along the Deseado Fault (DF). Note the trees (see arrows) that are tilted (or broken) along the fault scarp and then grow vertical to the left of the person for scale. This was likely due to localised tilting of the trunks during a recent surface rupture (likely < 500 years before present) along this shutter ridge, with the damaged trees then growing vertically. (**b**) Fault rocks within the Deseado Fault (DF) including gouge and breccia. These fault rocks are located along-strike (with a roughly W-E striking fault plane), parallel with, and within only a few hundred meters from the shutter ridge shown in A which demonstrates the bedrock faulting is responsible for the tectonic geomorphology found here. (**c**) Field photograph along the Magallanes Fault (MF) at the Lainez River site (Fig. 5) in Argentina along strike with the slip sites documented in the SfM model. Note the shutter ridge to the left of the person, which is present as an uphill facing scarp. (**d**) Sag pond along strike with the shutter ridge again from the Lainez site (Fig. 5). Sag ponds is located within a narrow (~50 m wide) damaged (at the surface) zone along the main MF. All photos were taken by the authors.

**Figure 8:**
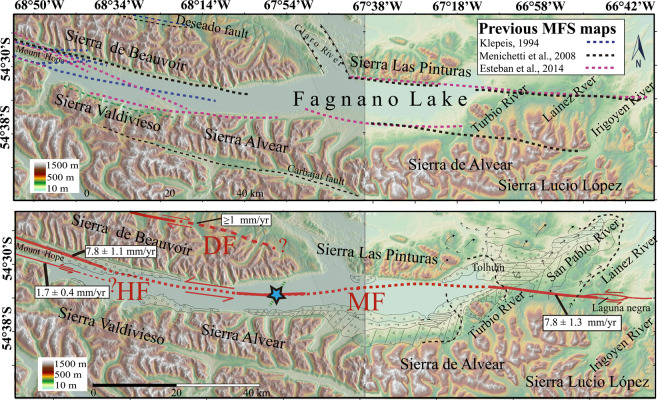
Upper map shows MFS mapping compilation in Lago Fagnano area of TdF by Klepeis (blue dotted lines)^5^; Menichetti (black dotted lines)^21^ and Esteban (pink dotted lines)^20^. Green dotted line indicate location for seismic profile in the Lake Fagnano and light blue star indicates location for a well defined MF fault zone observed in this seismic profile^11^. Bottom map show MFS mapping during this work based on field and remote sensing data observations. Red solid lines indicate locations for active faults reported in this study. Dotted red lines indicate locations for inferred fault continuations along Lake Fagnano. Geological offset measurements sites visited during this study are shown with black tick marks and boxes show long-term (Late Pleistocene) slip-rates calculated or inferred during this study from structures based on measured geomorphological markers offsets and regional dating^30,32^. Future work in TdF may demonstrate that faults shown in the upper map to be active. Base hillshade was generated with ESRI ArcMap v.10.3 software (under fair terms of use, https://www.esri.com/en-us/legal/copyright-trademarks)^58^ using a digital elevation model downloaded from ALOSPALSAR Global Radar Imagery with 12.5 m resolution (https://asf.alaska.edu/data-sets/sar-data-sets/alos-palsar/)^55^.

The MFS, from Cabo Froward (at the Strait of Magellan) east and crossing TdF is a well-defined strike slip plate boundary, with localized deformation accommodated along discrete sinistral sub-parallel crustal faults (Figs. 1 and 2) and are important sources of historical seismicity^9,12^. Based on our review of geologic mapping and a review of the literature along the MFS^4–6^ this plate boundary fault system has a long-term sinistral Late-Cenozoic slip rate of 5.4 ± 3.3 mm/yr (from 2.1 to 8.7 mm/yr), however there is large uncertainty as to the timing of onset. Further work here on the geology will provide better constraints on long term tectonics. In Chilean TdF, although we have calculated slip rates for the MF and HF, the DF’s slip rate is uncharacterised, however, we infer it to be at least 1 mm/yr (and perhaps higher) based on strong tectonic geomorphology including shutter ridges and deflected streams found in the field, and trees that appear to have tilted during the most recent event here (Fig. 7A,B; Supplemental Material). When comparing the nature and expression of the DF to other humid climate strike-slip faults we have witnessed in the field (e.g. New Zealand’s Hope fault and Alpine fault^35,36^), the strong tectonic geomorphology along the DF in Chile allows an inference of a slip rate of at least 1 mm/yr. Therefore, by combining our MF and HF slip-sites in the Chilean portion of TdF and including the at least 1 mm/yr DF slip rate inference provides a total Late-Quaternary geologic strike-slip rate across the MFS of at least 9.1 to 12.0 mm/yr. Our new Late-Quaternary (Late-Pleistocene) strike-slip rates of 7.8 ± 1.1 mm/yr (6.7 to 8.9 mm/yr, Chile) and 7.8 ± 1.3 (6.5 to 9.1 mm/yr, Argentina) were obtained for the MFS main strand (MF) based on regional Late-Quaternary glacial retreat timing (Fig. 6). The DF, (Fig. 1) ~15 km north Lake Fagnano^5^ has clear geomorphic evidence of recent fault ruptures. The deformed trees observed in the field (Fig. 7) along the DF (Figs. 1 and 8), are “Coihue de Magallanes” (*Nothofagus Betuloides)* which can live up to 500 to 600 years in Patagonia^37^, however Coihue trees on the northern side of Lake Deseado were dated using dendrochronology as being up to 340 years old (i.e. starting growth around 1680)^38^. The trees are located on a shutter ridge where the strike of the DF can be traced to fault rocks found in a nearby exposure demonstrating the bedrock faulting is impacting the geomorphology of the overlying Quaternary deposits (Fig. 7). Thus we can infer that the most recent event along the DF occurred post 1680 because these trees found on this shutter ridge are tilted and damaged (Fig. 7), specifically there are trees that are broken and growing diagonal from the surface. By combining the slip rates of three known active strands of the MFS (Fig. 8; i.e. MF, HF, DF) at this location in Chile gives a combined MFS strike slip slip rate of at least 10.5 ± 1.5 mm/yr. Are there active MFS faults north and parallel to the DF? This remains unknown. Interestingly based on GPS velocities and modelling^13^, the faults mapped south the HF are believed to not be active within onshore TdF. Being that the slip rate in Argentina is slightly slower at 7.8 ± 1.3 mm/yr suggests that perhaps other sub-parallel faults to the MF are likely accommodating slip there, perhaps the along the fault found at the southern end of Lake Fagnano^21^ (Fig. 8) which perhaps then connects with the HF. There the ranges of the Sierra de Alvear near the Turbio River (Fig. 8) appear to be displaced sinistrally across this structure^21^, thus this may help explain the potential slip discrepancy between the sites in Chile and Argentina. Ultimately, if there are other faults south of the HF or north of the DF that are accommodating modern deformation within the MFS, total Late-Quaternary geologic slip rates along this system could potentially be higher than 10.5 ± 1.5 mm/yr.

Geodetic models provide MFS slip rates that are within the same magnitude, however slightly slower, than the geologic rates we present here (6.6 ± 1.3 mm/yr^13^; 5.9 ± 0.2 mm/yr^14^; 9.6 ± 1.4 mm/yr^15^), however, our new geologic rates are consistent with the NUVEL-1 model^39^ which suggested the MFS should accommodate a minimum of 40% (or 8.8 mm/yr) of the rate of separation between South American and the Antarctic Peninsula (22 mm/yr). Nevertheless, any discrepancies between geodetic and geologic rates could be explained by choice of modeling parameters for the crust and/or the effects of the seismic cycle^40^. Although event location information has significant uncertainties due to a sparse seismic network, recent historical events (Fig. 1; two Mw 7.5+ events in 1949, Mw 7.0 in 1950, and Mw 7.2 in 1970) may have sufficient influence on the geodetic models to underestimate motions^40^. Did the historic events occur along the same fault, that is only along the main MF? Importantly, paleoseismology work focused on dendrochronology from trees found along the MF east of Lake Fagnano^26^, dated abrupt changes in tree rings (i.e. from concentric to asymmetric rings) in 1883 ± 5 and 1941 ± 10, which suggests two ruptures in close succession of this section of the fault. In New Zealand, along the plate boundary Alpine Fault, it was found using dendrochronology that trees within 15 km of the main fault zone^41^ were damaged by the Mw ~ 8 earthquake in 1717^42^. Thus we can assume that the historic events in ~1879 and 1949, where shaking was experienced in Southern Patagonia, were due to ruptures along the MF. Young tectonic geomorphology along the MF (Fig. 7) including sag ponds and shutter ridges could be targeted for better constraints on timing of pre-1949 and pre-1879 events to better characterise timing and recurrence of events. Or did events occur along both the MF and/or HF and DF during this same time period (Figs. 7 and 8)? Along the DF, there also damaged trees that appear to be growing diagonally, with a clear kink in the tree trunk where they then grow vertically, this is consistent with damage to the trunk of the trees during surface faulting (and tilting of the trunks away from vertical), and then the trees growing vertically post-event (Fig. 7)^42^. Although further work in the field should unravel these inferences regarding tree damage along the DF, based on similar observations of damaged trees located growing on and near scarps of the strike slip Alpine Fault in New Zealand^35,42^, it appears that trees along the DF (Fig. 7) were tilted and damaged by a DF rupture and earthquake in the lifetime of this grove of Coihue trees (i.e. post 1680)^38^. Despite these uncertainties, did more than one fault in the MFS rupture together like recent modern events demonstrate can occur (e.g. the 2016 Kaikoura earthquake in New Zealand^43^)? Or do they operate independently? Although our new data provide important constraints on the rates, there are still a number of open ended key questions here specifically related to recurrence of events and timing most recent events on these faults. Because the faults in the MFS are fast slipping and a major source of crustal seismic hazard, these faults should be avoided to reduce fault rupture hazards (both onshore and in the Canals of southern Patagonia) in addition to being important sources of strong ground motions and coseismic hazards (e.g. landslides, liquefaction, rockfall). Since TdF is a hydrocarbon-producing region, understanding exactly where MFS active faults are located is also essential to reduce the potential for triggered seismicity due to contemporary fracking during oil and gas extraction. As much of the MFS is submarine either in the Atlantic Ocean or within the fiords of Chile, future marine geology investigations should address these unknowns.

Since the MFS is fast slipping, and long-lived (since at least 6 Ma and up to 20 Ma), and apparently narrow, what does it tells us about strike slip plate boundaries? Modern active and steeply dipping strike slip faults of the MFS cut the contractional structures of the Magellan Fold and Thrust belt^44,45^ (Fig. 1). However extensive regional glaciations (Fig. 6) combined with extensive vegetation (and much of the MFS being submarine) conceal faults here. Modeling suggests that deformation along MFS faults are focused around a narrow ~ 40 km-wide zone around the MF^14^. Our mapping, fieldwork, and analysis suggest that at least three sub-parallel sinistral strike slip structures within the MFS, from north to south, the DF, MF, and HF, accommodate most of the ongoing active strike slip plate boundary deformation here in a very narrow zone that is <20 km wide at the surface (Figs. 1 and 8). Based on observations from most onshore plate boundaries, generally deformation is partitioned along faults on both sides of the master faults (e.g. Alpine, San Andreas, and MF plate boundary faults), and thus slower slipping faults (although still active and accommodating plate boundary deformation) should be expected south of the HF and north of the DF and with less clear expression in the tectonic geomorphology due to slower rates. South of the HF, in the Beagle Channel (Fig. 1), there is proposed neotectonic activity of faults there based on relative altitude differences of marine terraces^46^, and presence of post Early Cretaceous strike slip faults that are identified in the field, however if these faults are indeed active requires future attention. Compared with the width of surface deformation along other onshore active continental strike-slip plate boundaries, for example the North American/Pacific Plate along the San Andreas Fault^47^ (up to 200 km) and the Marlborough Fault System in New Zealand^48^ (up to 150 km), the MFS’s 30-50 km width in TdF is very narrow and localised from the Strait of Magellan to east (Fig. 1). This makes the MFS a candidate for an end member within plate tectonics, and perhaps one of the narrowest and most simplistic strike slip plate boundaries on Earth (Supplemental Material). The MFS well-developed geometry, simplicity (straight and localised) and remarkable length, could suggest a low ratio of small to large earthquakes due to smoothing along these faults and thus magnitude-frequency distributions derived from seismology may not reflect the actual seismic hazard^49^. The limited period of instrumental recordings along the MFS, when compared with results from California^50^, suggests that the MF may not be suitably described by the Gutenberg-Richter relationship^51^ between major earthquakes; and that the MF does not have frequent moderate-sized earthquakes, but instead remains locked with major stress drops only occurring during large earthquakes and associated aftershocks.

West of TdF near Cabo Froward (Fig. 1; Supplemental Material) faults within the MFS appears to spread out, and widen at the surface to >100 km, and curve (i.e. change strike) towards the north forming a more diffusely distributed, and thus wider fault system than from TdF and east^19^. We speculate that this could due to the influence of the subduction zone (Fig. 1) where the MFS Plate Boundary is influenced by the complexities and heat flow found near the triple junction and/or perhaps related to effects of the Patagonian bend^16^. Another possibility is that there are extensive sub-vertical pre-MFS basement crustal faults with a similar array to that which we see today and which reactivated when the MFS system initated^2,45^. However, the most likely case is that the MFS system becomes more slip partitioned between the offshore subduction of the Antarctic Plate in the northwest (with more widely-spaced crustal faults) and the concentrated and narrow onshore strike slip faulting within in the MFS found in the area around TdF. We thus speculate that the MFS west of TdF is perhaps somewhat analogous to the Marlborough Fault System in New Zealand which becomes more slip-partitioned as the crustal plate boundary encounters the Hikurangi subduction zone, versus being more narrow and localised along the Alpine Fault^36,43,48^. However based on our fieldwork and mapping when compared with the literature, from TdF to the east, it appears that the MFS could be one of the narrowest, straightest, and simplest strike-slip plate boundaries on Earth^3^. As slip-rates can change over long-time scales^47^, future fieldwork and geochronology will better constrain timing of MFS initiation and thus better constrain the long-term slip rate, neotectonic behaviour including seismic hazard, and Late-Cenozoic regional tectonics.

## Methods

To better understand long-term Late-Cenozoic geological separations along the MFS we reviewed the literature and previous geological mapping^5,44,52^. The amount of the long-term strike slip along the MFS was estimated by the correlation of stratigraphic limits and analyzing regional structures in relation to the lithologic units mapped in the region^5,22,44,53^ (Table S1). Mapping of faults along the MFS was undertaken in Google Earth^54^ and a 12.5 m/pix resolution digital elevation model (DEM) of the region derived from ALOSPALSAR Global Radar Imagery data (Acquisition date: 2010-12-30, 2011-01-11)^55^ to identify potential fault traces and displaced Quaternary features. Remote mapping was then field validated. Additionally, air-photos were collected in the field with a DJI Phantom 4 drone at sites identified during desktop mapping (Supplemental Material). In Chile we screened from the Beavouir Range to the northern edge of Lake Fagnano to best identify MFS fault traces (Fig. 2). These aerial photographs were modelled using Agisoft Standard Photoscan Pro 1.3.2 (2018)^56^ in order to develop Structure from Motion (SfM) site models^57^ including 3D DEMs (which include vegetation and thus could be referred to as Digital Surface Models (DSM)), and full colour orthophotos (<1 m/pixel; see Supplemental Material). We note this process was iterative, because drone flights allowed for identification of additional field sites. These data were imported into ArcMap ArcMap 10.3 software for spatial analysis^58^. During fieldwork, characterisation of deposits and tectonic geomorphology were also documented.

Using the SfM models, faults within the MFS were located as an idealized planar traces, and displaced geomorphic markers were observed along those traces^59^. After the fault was located and traced as an idealized planar fault trace, displaced geomorphic markers (e.g. river channels, terrace risers, displaced hillslopes) were documented and the offsets were measured^59,60^. These offset clusters are interpreted as the cumulative slip associated with fault ruptures that occurred post-ice retreat since the fault scarps are formed in Late-Quaternary glacial deposits. These markers were inspected in detail (both in the field in the models) to avoid landforms that possibly were modified by other reasons, for example, channel deflection or meanders, or due to anthropogenic reasons (i.e. fences, tree felling for timber or firewood). To determine the horizontal offset in each marker, an appropriate projection of the piercing lines marker both sides the fault onto the linear fault trace^60^ Additionally, fault-parallel elevation profiles were derived using Spatial Analyst within the ArcMap 10.3 software^58^, where the offset were defined as clear variation within the models or the horizontal distance needed for back-slip the markers along both sides the fault trace. For the interpolation of the markers and profiles, the original morphology for the feature or landform was estimated. A range of plausible offsets amounts was given as a minimum and maximum for each marker related to the range of values that credibly restored the original morphology in a specific site^35,60,61^. The acceptable offset range is dependent on the scale of geomorphic features versus magnitudes of offset, the clarity of landform features and the precision of measurement tools^61^. Linear features that could be traced to the fault on both sides have smaller uncertainties that those features that needed to be projected obliquely^59^. This was designed to measure the largest number of representative offset markers at each site with the aim of constraining the uncertainties in the measurements.

Uncertainties related to the offset measurements due to the quality of the offset value (based on the reliability and accuracy of the feature that is being matched across the fault) was also documented^59^ (from A to C from high to low degree of confidence, respectively; see Supplemental Material). An appropriate projection of the piercing points were made to the faults with horizontal distances measured. Possible post-rupture modifications by erosion and deposition were considered to estimate of the original morphology of the features. A range of plausible offsets amounts was given as a minimum and maximum for each marker associated to the range of values that credibly restored the original morphology in a specific site^59^. Although the resolution of the DEMs were generally a 1 m for these sites, we ascribe uncertainties (based on creek modifications and vegetation) of ~3 m to 10 m per site measurements to account for potential erosion^62^. The Late-Quaternary slip-rates were obtained by combining the measured offsets derived from the SfM models (with uncertainties) combined with published ages (including uncertainties) from surfaces and deposits (Figs. 2–6). A mean value with its standard deviation were calculated to determinate the minimum and maximum slip-rate for the site as a whole. A closure criterion was used to estimate the overall relative slip along the fault system in case that two fault traces are assumed as parallel, the sum of the slip rates are considered approximately match the overall slip rate^40^.

## Supplementary information

Supplementary Materials File.

Data S1 - MFS SfM dataset.

Data S2 - MFS SfM dataset.

Data S3 - MFS SfM dataset.

Data S4 - MFS SfM dataset.

Data S5 - MFS SfM dataset.

Data S6 - MFS SfM dataset.

Data S7 - MFS SfM dataset.

## Data Availability

All data generated or analysed during this study are included in this published article (and its Supplementary Information files).
