# Supplementary material for: Slip rates along the narrow Magallanes Fault System, Tierra Del Fuego Region, Patagonia
Source: Sci Rep. 2020 May 18;10:8180. doi: 10.1038/s41598-020-64750-6 (PMC7235261; doi:10.1038/s41598-020-64750-6)
Supplement: Supplementary file 1 — Supplementary Materials File. [file 41598_2020_64750_MOESM1_ESM.pdf]

Supplemental Material

Slip rates along the narrow Magallanes Fault System, Tierra Del Fuego Region, Patagonia

Francisca B. Sandoval and Gregory P. De Pascale*

Departamento de Geología, FCFM, Universidad de Chile, Plaza Ercilla 803, Santiago de Chile

*Correspondence to: snowyknight@gmail.com

This PDF file includes:

Additional methods, models, review of the MFS (with references) and observations

Figures S1 to S19

Tables S1 to S8

Captions for supplemental data files S1 to S19

Supplemental data files (Acrobat 3D pdf files) in support of this manuscript include the following:

Data S1: MF in Chile 3D SfM model (3D pdf)

Data S2: Western Azopardo Valley, near Caleta María 3D SfM model (3D pdf)

Data S3: Central Azopardo Valley 3D SfM model (3D pdf)

Data S4: Eastern Azopardo Valley near Lake Fagano 3D SfM model (3D pdf)

Data S5: Turbio River Valley in Argentina 3D SfM model (3D pdf)

Data S6: Western Turbio River Valley in Argentina 3D SfM model (3D pdf)

Data S7: Lainez River, Argentina 3D SfM model (3D pdf)

Supplemental Material Overview

This file includes additional figures, tables, text and data files in support of the results and discussion in the main paper. Additional figures on glacial history (Figures S1, S2), seismicity (Figure S3), additional drone-acquired airphotos, SfM models, and field photography (Figures S4-S12), a seismic profile showing the MFS disrupting lakebed sediments within Lake Fagnano (Figure S13), drone camera locations and error estimates in in the SfM models (Figures S14-S18), and a image showing relative width at the surface of plate boundary deformation along four of Earth's major strike slip plate boundaries (Figure S19). Additional tables (Tables S1-S8) cover background on the MFS slip, slip rates, model parameters, and slip rate details derived from this study. Additional references not already referenced in the main paper, are referenced below are listed towards the end of this supplemental information. All field photographs were taken by one of the two authors and are original works.

Additional Southern Patagonia glacial history:

Figure S1

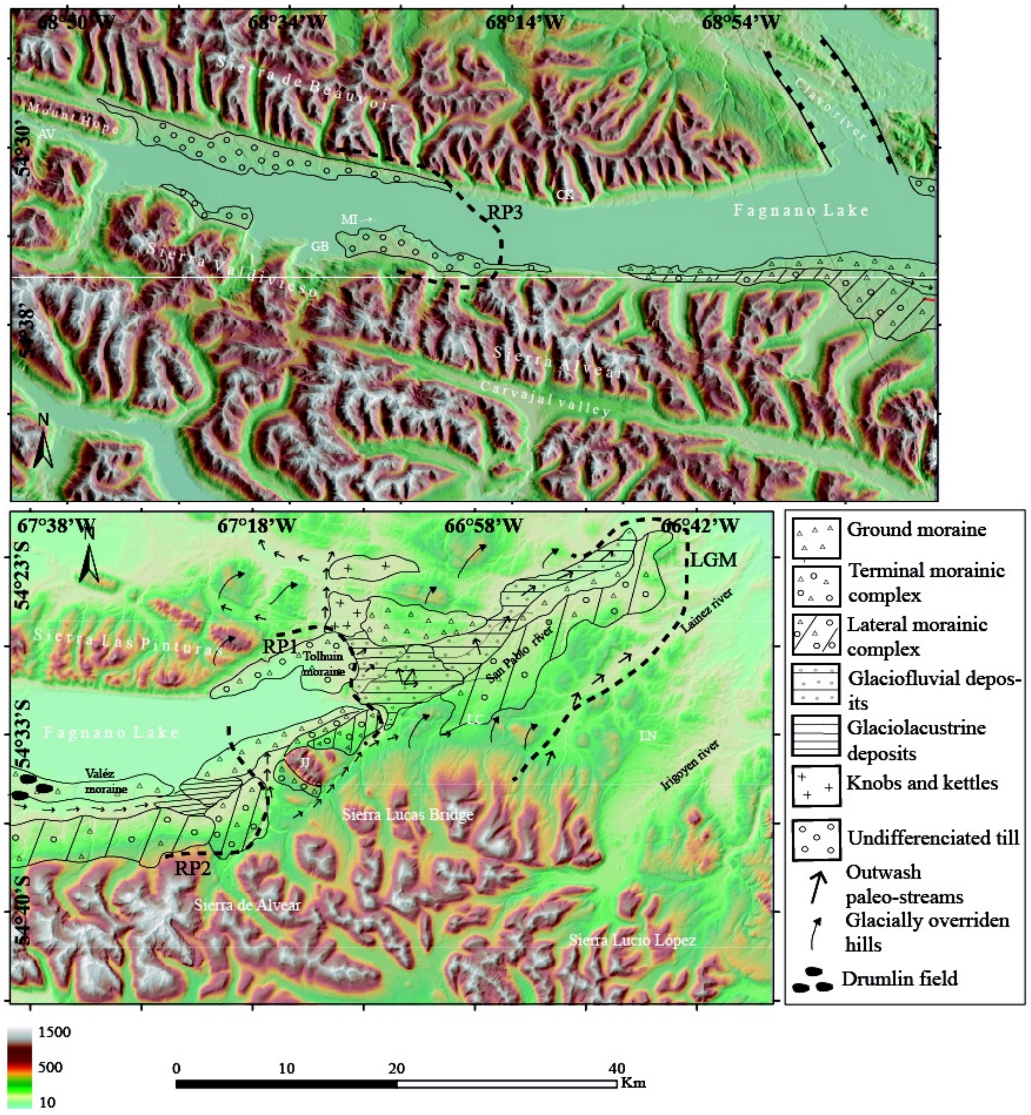

Compilation of glacial landforms and deposits in Lake Fagnano area, Patagonia. Base map corresponds to the high-resolution terrain-corrected ALOSPALSAR Global Radar Imagery with 12.5 m resolution illuminated DEM (Acquisition date: 2010-12-30, 2011-01-11). Doted black lines indicate LGM maximum extension and Recessional Phases. Elevation in m. RP1-RP3: Recessional Phases 1 to 3 boundaries (after Coronato et al., 2009). LN: Laguna Negra bog, LC: La Correntina camping, JJ: Jeu-Jepen hill, MI: Martínez Island, GB: Grande Bay, CK: Kranck Hill, AV: Azopardo Valley. Base hillshade was generated with ESRI ArcMap v.10.3 software (under fair terms of use, https://www.esri.com/en- us/legal/copyright-trademarks) using a digital elevation model downloaded from ALOSPALSAR Global Radar Imagery with 12.5 m resolution (https://asf.alaska.edu/data-sets/sar-data-sets/alos- palsar/).

Figure S2

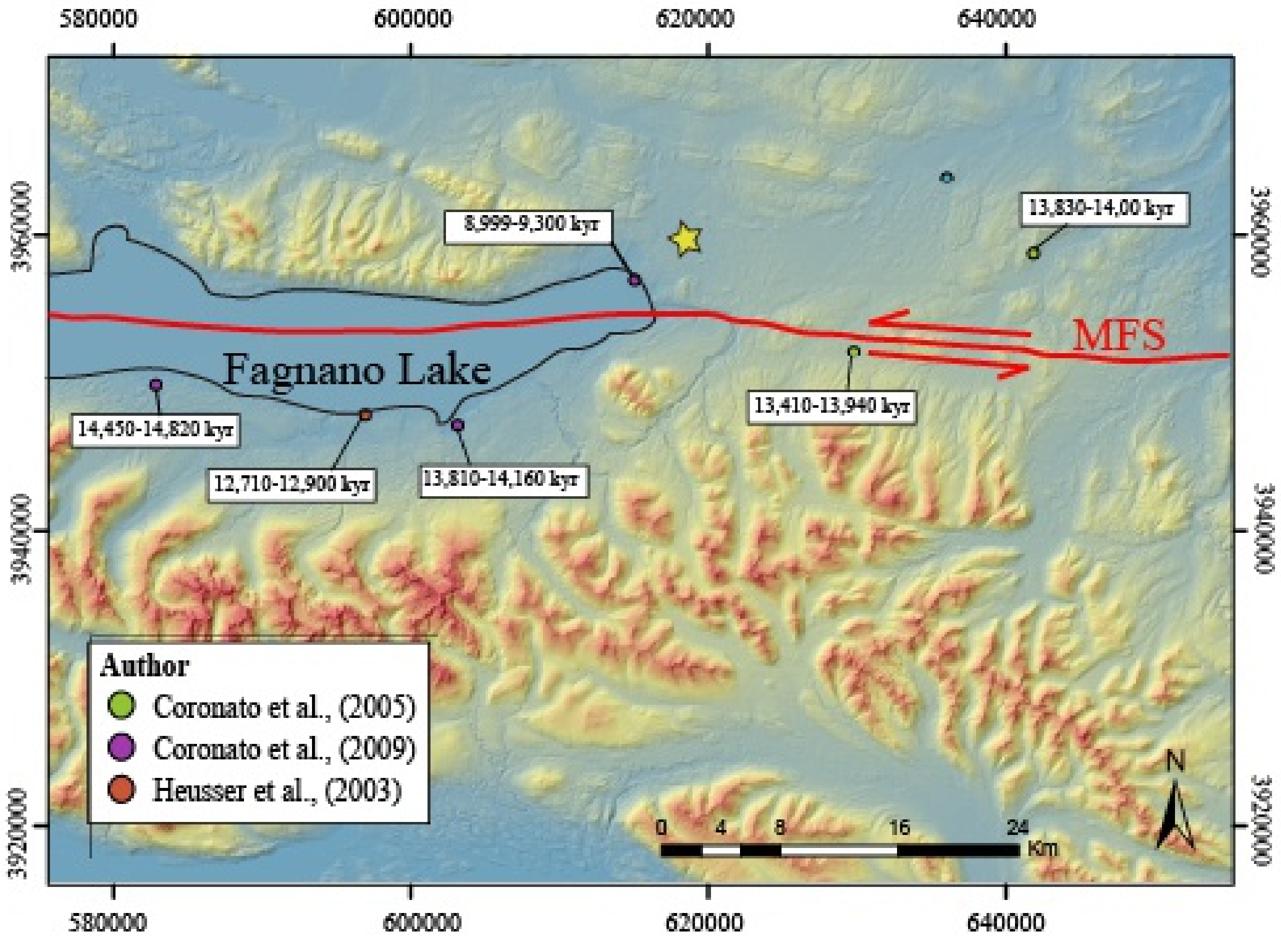

Peat bog radiocarbon dating by previous authors in the Argentina portion of Lake Fagnano (Coronato et al., 2005; Coronato et al., 2009; Heusser et al., 2003) Yellow star is the town of Tolhuín. The base map ALOSPALSAR 12.5 m DEM. Red solid lines indicates main MF trace within the MFS. Base hillshade was generated with ESRI ArcMap v.10.3 software (under fair terms of use, https://www.esri.com/en- us/legal/copyright-trademarks) using a digital elevation model downloaded from ALOSPALSAR Global Radar Imagery with 12.5 m resolution (https://asf.alaska.edu/data-sets/sar-data-sets/alos- palsar/).

Additional background regarding neotectonic parameters and seismicity along the MFS

Table S1: MFS long-term cumulative offset with its respectively kinematics.

Site	Lateral offset	Vertical Offset	Kinematics	Reference
Contact between Upper Cretaceous-Paleocene rock units in an area southeast TdF	48 km ± 20	none	Sinistral	Torres-Carbonell et al, 2008
Near Argentina-Chile limit in Mount Hope area, in a contact between Upper Jurassic and Lower Cretaceous	25 km of cumulative offset of each recognized fault, and only 15 km considering regional offset.	none	Sinistral with an extensional component	Klepeis, 1994
Offset in a previous fault zone both sides of the MFS (Río Candelaria Río Claro y Estancia La Correntina-Bahía Sloggett)	55 km	none	Sinistral	Rosello et al, 2005
In Argentinean side of the Isla Grande, from the contact between stratigraphic units both sides of the fault.	20- 30 km	none	Sinistral	Olivero & Martinioni, 2001
Apparent offset in the western margin of the Patagonian Batholith	80 km	none	Sinistral	Winslow, 1982

Table S2: MFS Modeled slip rates found in the published literature.

Slip rate	Reference	How the slip-rate was estimated
6.6 ± 1.3 mm/yr	Smalley et al 2003	Independents set of 20 GPS stations in the central portion of the MFS, assuming a vertical slip fault model with 15 km locking depth.
5 mm /yr	Pelayo & Wiens, 1989	Slip vectors from source parameter inversion of waveforms and amplitudes for eight strike-slip and thrust faulting events along the Scotia plate. According to the author, the results are poorly constrained.
4.4 ± 0.6 mm/yr	Mendoza et al., 2011	Repeated geodetic observations since 1993 of a GPS regional network of 29 sites in central and southern Tierra del Fuego Island in Argentina.
5.9 ±0.2 mm/yr	Mendoza et al., 2015	Block model of the MFS fault trace using interseismic velocities from 48 sites, obtained from field measurements spanning 20 years, considering two blocks (SAM and SCO Plates) and three main fault traces (Irigoyen, Turbio and Lake Fagnano valleys)
9.6 ± 1.4 mm/yr to 8.9 ± 1.2	De Mets et al., 2010	MORVEL model from the western to the eastern NSR based on geodetic observations and spreading rates
7.0 ± 3.5 mm/yr	Thomas et al., 2003	Using a combination of slip vectors, spreading rates and azimuths presented a solution for the Scotia plate motion. Slip rates for the NSR and the SSR were obtained by closure.

Additional background on regional seismicity in Tierra Del Fuego (TdF)

Table S3: Historical seismicity of TdF (ISS – ISC: Catalogue of the International Seismological Centre, Berkshire, Great Britain. (http://www.isc.ac.uk), UNLP (Universidad Nacional de La Plata, Jaschek et al., 1982*)*

Date	Reference	Time	Lat	Long	Depth	Magnitude
**02/02/1879**	Lomnitz, 1970	03:30 (local)	-	-	-	M7.0-7.5
**17/12/1949**	Jaschek et al., 1982	00:12 (local)	54.24°S	69.03°W	10 km	Ms7.8
**17/12/1949**	Jaschek et al., 1982	15:52 (local)	53.89°S	69.67°W	10 km	Ms7.5
**30/01/1950**	Jaschek et al., 1982	00:56 (UTC)	53.5°S	71.5°W	10 km	M7.0
**15/06/1970**	ISC	11:14 (UTC)	54.467°S	64.499°W	10 km	M7.2

Despite of the limitations in terms of spatial distribution and number of seismic stations in TdF, some authors recorded the instrumental seismicity over periods no longer than 2 years, thus location uncertainties are high (Febrer et al., 2000; Sabbione et al., 2007; Buffoni et al., 2009). Since 1999 a seismological network of 5 stations in the Argentine portion of TdF was installed and >300 earthquakes spatially related with the MFS were registered (Buffoni et al., 2009). The magnitudes of these events are in general low to moderate (Mw ~2 to <5) (Winslow 1982, Pelayo & Wiens 1989; Febrer et al., 2000; Sabbione et al., 2007, Buffoni et al., 2009). Other cluster of events is near the Beagle Channel and in the southernmost Fueguian archipelago. Offshore to the east of TdF some epicenters are related to the North Scotia Ridge, while to the south are associated to the subduction in southern limit of the Chilean trench and in the Shackleton fracture zone.

Table S4: Recurrence intervals determined on the MFS in previous works

Reference	Evidence or how this was determined	Recurrence interval
Costa et al., 2006	Fault slip by the changes in trench sediments along a secondary fault trace	2.0 ka
Waldmann et al., 2011	Slope failure and megaturbidite deposits in sub-basin of Lake Fagnano	0.35-0.85 ka
Bonorino et al., 2012	Coesismic faults in two strand plains north of the Strait of Magellan in response to deep-seated faulting	0.9 ka
Bonorino et al., 2012	Coseismic uplift of marine littoral deposits along the Beagle Channel, in steps 1–2 m in height, an offset that could be accounted for by earthquakes of ca M = 8	1.2 ka
Smalley et al., 2003	Based on the slip rate of the fault and a 5 m of offset for the 1949 earthquakes	0.75 ka

Figure S3

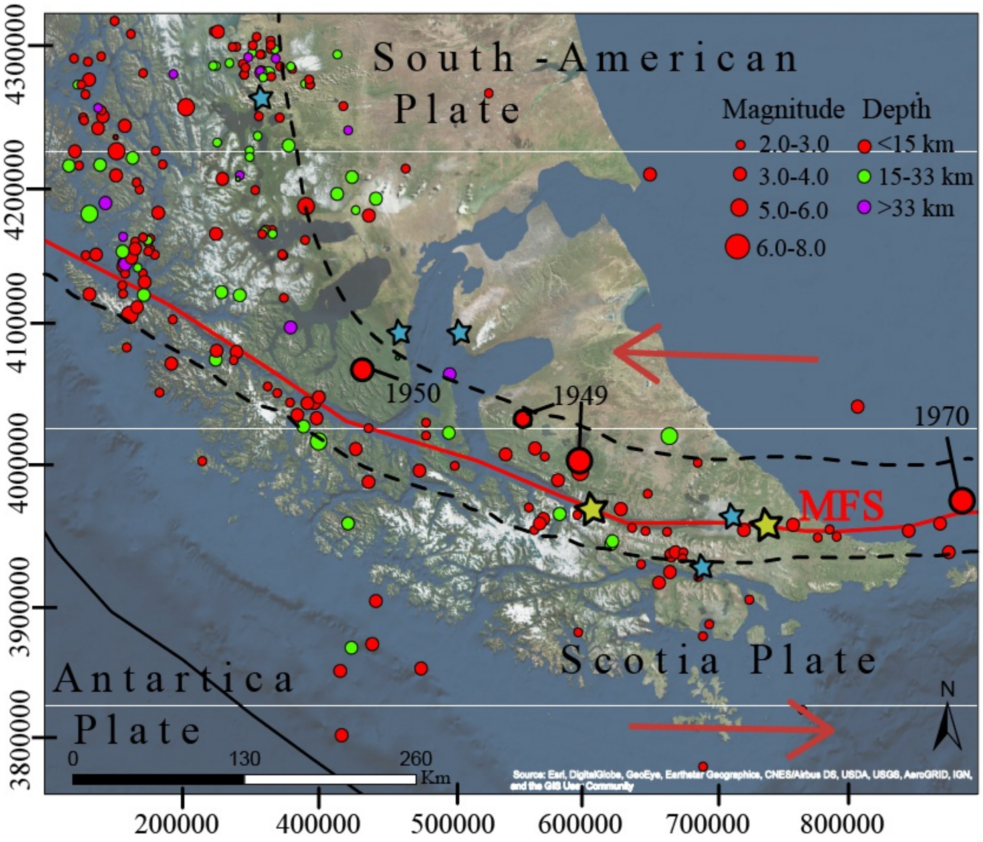

Seismicity in Southern Patagonia (ISS – ISC: Catalogue of the International Seismological Centre, Berkshire, Great Britain. (http://www.isc.ac.uk), UNLP (Universidad Nacional de La Plata, Jaschek et al., 1982). Epicenters from east to west: Mw 7.8 1949 (10 km), Mw 7.5 1949 (10 km), Mw 7.0 1950 (15 km) Yellow stars feological slip-rate measurement sites in Chile (west) and Argentina (east). Light blue stars indicate urban centers (Puerto Natales, Punta Arenas and Porvenir in Chile and Tolhuín and Ushuaia in the east in Argentina). Base image was generated with ESRI ArcMap v.10.3 software (under fair terms of use, https://www.esri.com/en-us/legal/copyright-trademarks).

Additional Methods

Digital air photo acquisition: After a ground reconnaissance and examining the available aerial and topographic data, together with previous markers prospects examination, the optimum sites were chosen where reliable geomorphic features were offset by the MFS. Thousands of pictures were taken in 10 flights in Chilean portion of TdF Island (Figure S6) made by using the DJI Phantom 4 Quadcopter (a.k.a Unmanned Aerial Vehicle (UAV) or Drone). Stops were made along the route from the northwestern shore of Lago Fagnano, until Caleta María Bay by the Azopardo River Valley. In Argentina, 11 flights were made in Argentinean territory using the Drone along the ~E-W path of the fault trace from the eastern shore of Lago Fagnano until about 20 km at east, near Estancia La Correntina (Figure S5).

Figure S4

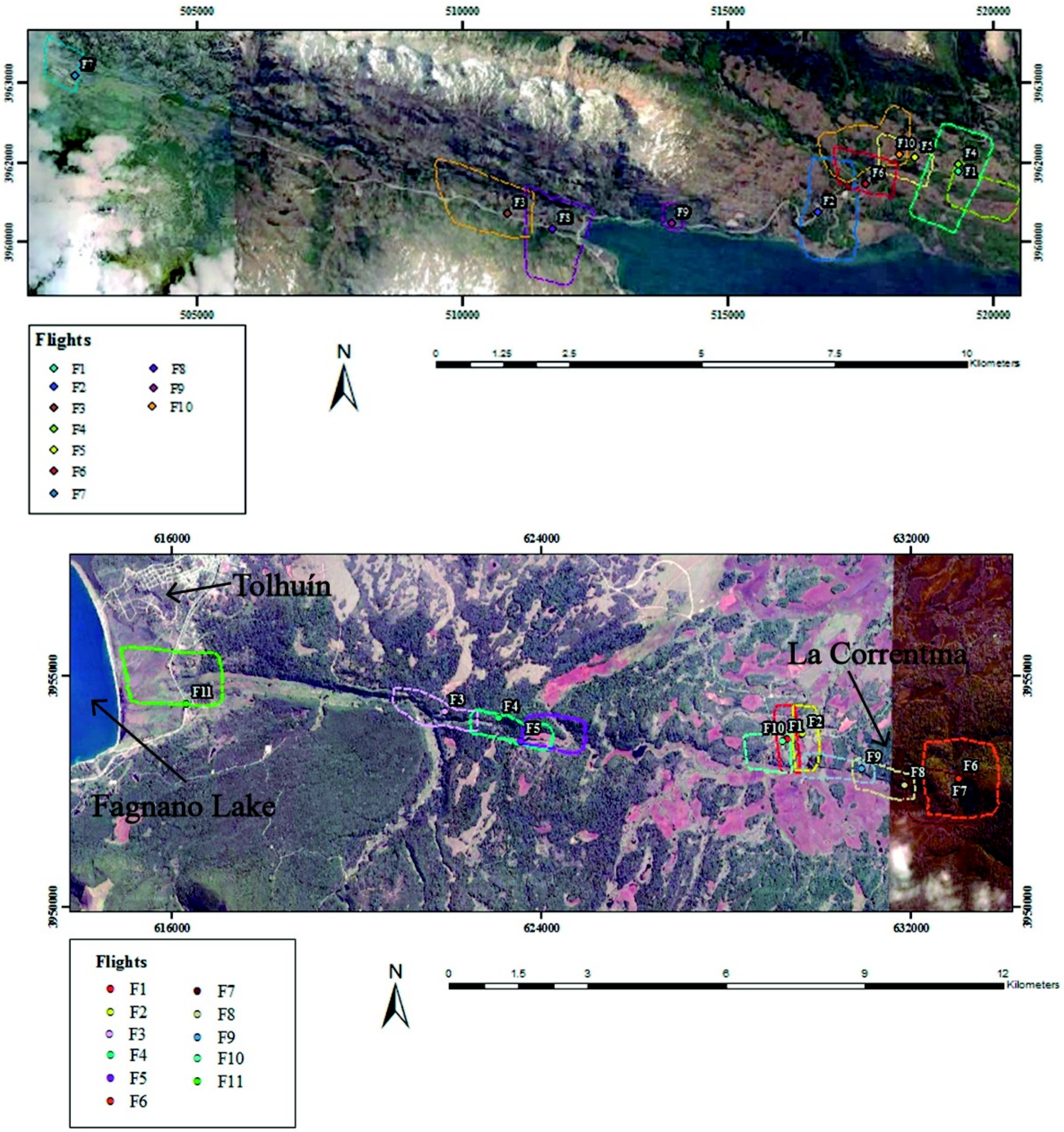

a) Locations of the drone flights in Chile. b) Locations of drone flights in Argentina. The colored point indicates the location of the drone pilot, dashed lines indicate the maximum extension of the flight area. Note that flights overlapped to obtain better coverage and model unity. Base image was generated with ESRI ArcMap v.10.3 software (under fair terms of use, https://www.esri.com/en- us/legal/copyright-trademarks).

Drone-acquired Structure from Motion (SfM) photogrammetry and geomorphic models

Digital photogrammetry permits construction of high resolution 3D digital elevation models (DEMS) from several 2D images acquired through remote controlled drones (Kurz et al., 2011; Johnson et al., 2014). This is done using stereoscopic methods which requires overlapping between pairs of images of the same scene from 60% laterally and 30% longitudinally (Duelis, 2015). Structure for motion (SfM) methods is one form of photogrammetry, which uses motion along a set of overlapping photos to obtain the 3D images, the topography and field geometry are automatically solved by an iterative process (Johnson et al., 2014). Subsequently, the recorded scene is analyzed with a SfM algorithm, which estimates the camera motion and creates a 3D point cloud of the static image (Kurz et al., 2011). This point cloud can then be used to generate a DEM that can be used in a GIS software package.

In the field we used a DJI Phantom 4 Quadcopter (i.e. UAV or Drone). At each site, an average of 250 photos per flight were acquired. The camera has a resolution of 4000 x 3000, a focal length of 3.61 mm and pixel size of 1.56 x 1.56 µm. Since we were working in an area with limited details, many of the sites were flown at a reconnaissance level to screen of MFS fault traces. These preliminary flights were then used to design flight lines to capture photographs along the fault targets. After each flight, the photos were downloaded, loaded into the photogrammetric software *Agisoft Standard Photoscan Pro 1.3.2* version build 4205, in a Mac OS 64 platform. This software, recognizes matching points between the images and through a comparative recursive process determines the geometry of the surface and the relative location of the cameras (Johnson et al., 2014). This base algorithm can be divided in two stages: the first approximation of the image's analysis and a resolution of the 3D space by a least square regression in no linear models. Based on the estimated camera position, the software calculates depth information for each point to be combined into a single dense point cloud. After dense point cloud is

built it is possible to generate a surface model or polygonal mesh obtaining the 3D model, on which is possible to make a texturizing process to capture the textural features on the smooth surface. For this, an orthophoto of the model is projected on the mesh surface from a specific view to obtain a site-wide high resolution image for mapping (Johnson et al., 2014).

The result was a total of 4 models in Chile and 3 models in Argentina. The corresponding high-resolution digital elevation models and hill shades were developed and used within 2D (ArcMap 10.3). The aim of these was the mapping of geomorphic features and landforms in the sections of interest with a much greater accuracy than is possible through available free DEM's (ALOSPALSAR Global Radar Imagery 12.5 m), in addition to a considerable better visualization of the 3D surface.

Figure S5

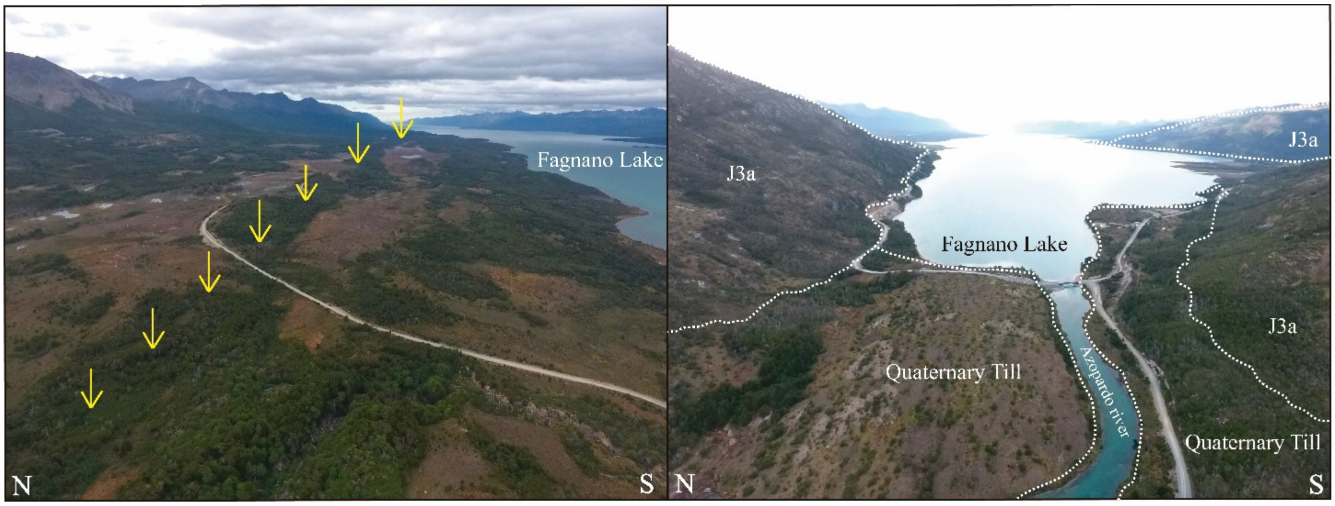

Aerial drone images in the northwestern shore of Lake Fagnano in Chile. a) Aerial photography of the northeastern shore of Lake Fagano, yellow arrows indicate the main MF in this area. b) View to the east in Azopardo Valley, bounded on the north and south by Jurassic volcaniclastic rocks of the Beauvoir Fm. (Caminos et al., 1981), a lobular till deposit entrenched to the south by the Azopardo River is cut and left-lateral offset by the HF. Original drone photography collected by the authors.

Table S5: Offset measurement synthesis for the MF in Chile, along the northwestern shore of Lake Fagnano, Chile.

**Chile - Site**	**Projection 1**	**Projection 2**	**Projection 3**	**Profile**
**Confidence degree**	A	A	B	A
**Offset (m)**	95 ± 5	85 ±5	90 ± 5	80± 5
**Geomorphological marker description**	Right limit of the channel	Left limit of the river	Thalweg of the channel	River channel back-slip
**Age max = 12.5 ka, Age min = 10.2 ka**				
**Slip-rate 7.8±1.1 mm/yr (6.7 to 8.9 mm/yr)**				

Figure S6

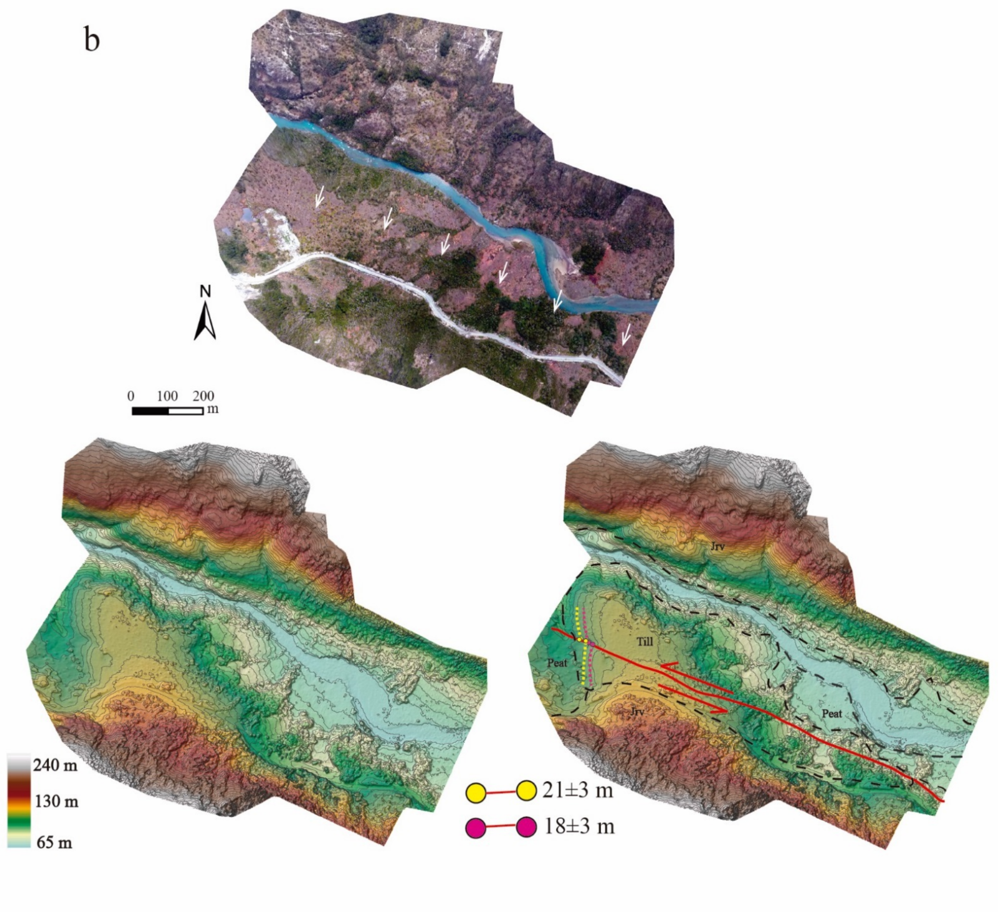

SfM models from the HF rupture in the center of the Azopardo Valley in Chile. In the ortophoto (top) the fault trace is delineated by vegetation and left-lateral offset a glacial deposit. Qal: recent alluvial deposits, Till: undifferentiated till, Jrv: Jurassic volcaniclastic rocks from Lemaire fm. (Caminos et al., 1981). Solid red lines indicate the fault trace. 5 m contours. SfM models were generated using the Agisoft Standard Photoscan Pro 1.3.2 (2018) (https://www.agisoft.com). DEM was processedwith ESRI ArcMap v.10.3 software (under fair terms of use, https://www.esri.com/en-us/legal/copyright-trademarks).

Figure S7

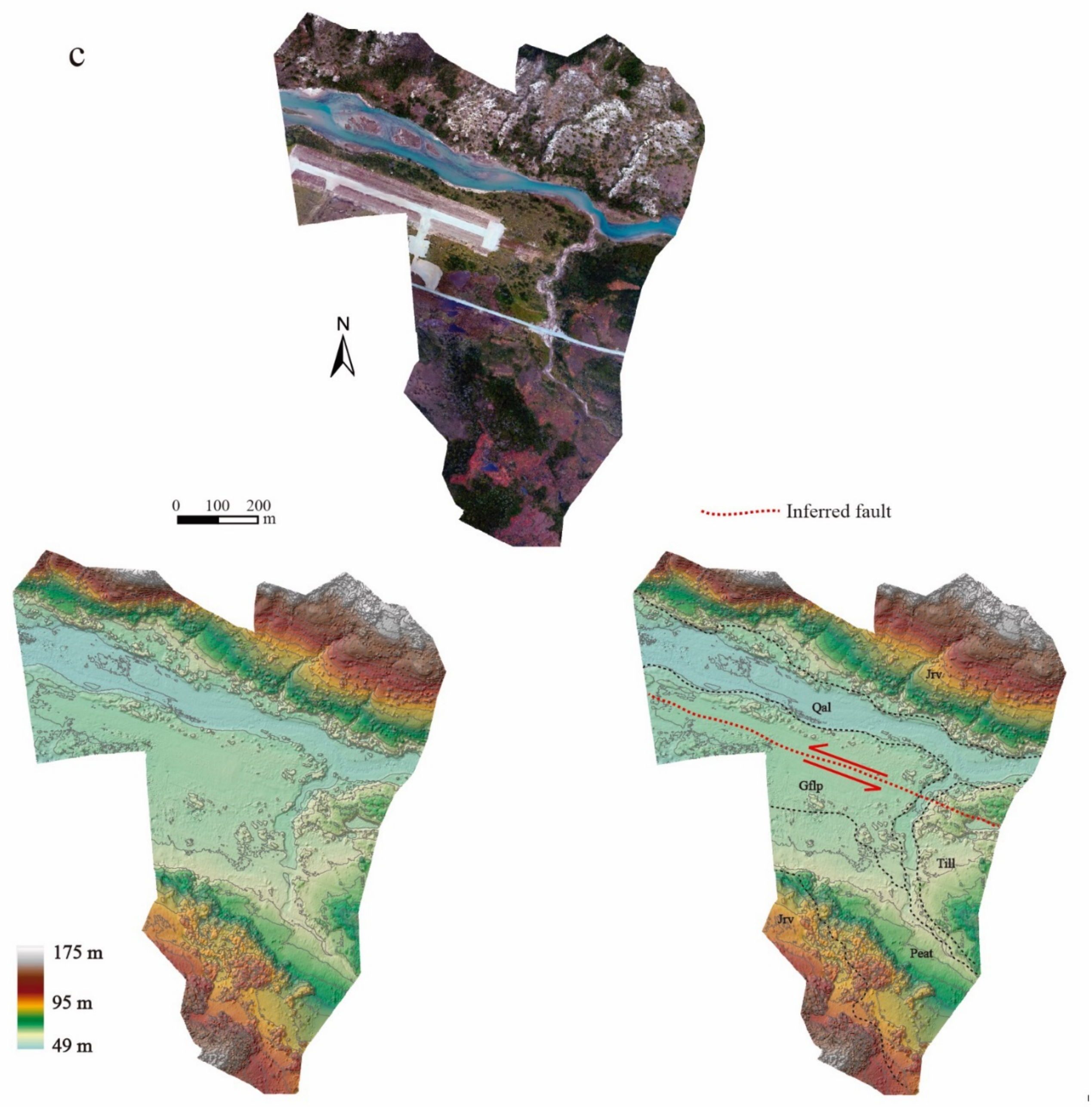

Westernmost models in Azopardo Valley, the fault rupture is not observed, probably because it is covered by peat, and recent deposits with flood plain morphology (and only a few m above sea level. The HF trace would continue parallel to the airstrip in Caleta María area. For figures locations see Figure 2.a. Qal: recent alluvial deposits, Till: undifferentiated till, Jrv: Jurassic volcaniclastic rocks from Lemaire fm. (Caminos et al., 1981), Qflp: Quaternary glaciofluvial plain. Dotted red line is inferred fault continuation at west. 5 m contours. SfM models were generated using the Agisoft Standard Photoscan Pro 1.3.2 (2018) (https://www.agisoft.com). DEM was processedwith ESRI ArcMap v.10.3 software (under fair terms of use, https://www.esri.com/en-us/legal/copyright-trademarks).

Table S6: Offset measurement synthesis for the Hope fault (HF), located in the northwestern shore of Lake Fagnano, Chile.

<u>Chile-Azopardo Valley</u>	**Projection 1**	**Projection 2**	**Projection 3**	**Projection 4**
**Confidence degree**	A	A	A	A
**Offset (m)**	18 ± 2	23 ± 2	16 ± 3	21 ± 3
**Geomorphological marker description**	180 Contour, in glacial deposit next the lake	185 Contour, in glacial deposit next the lake	112 Contour, in glacial deposit in the central eastern portion of the valley	112 Contour, in glacial deposit in the central eastern portion of the valley
**Age min = 10 ka**				
**Age max =12.5 ka**				
**Slip-rate 1.7±0.4 mm/yr (1.3 to 2.1 mm/yr)**				

Figure S8

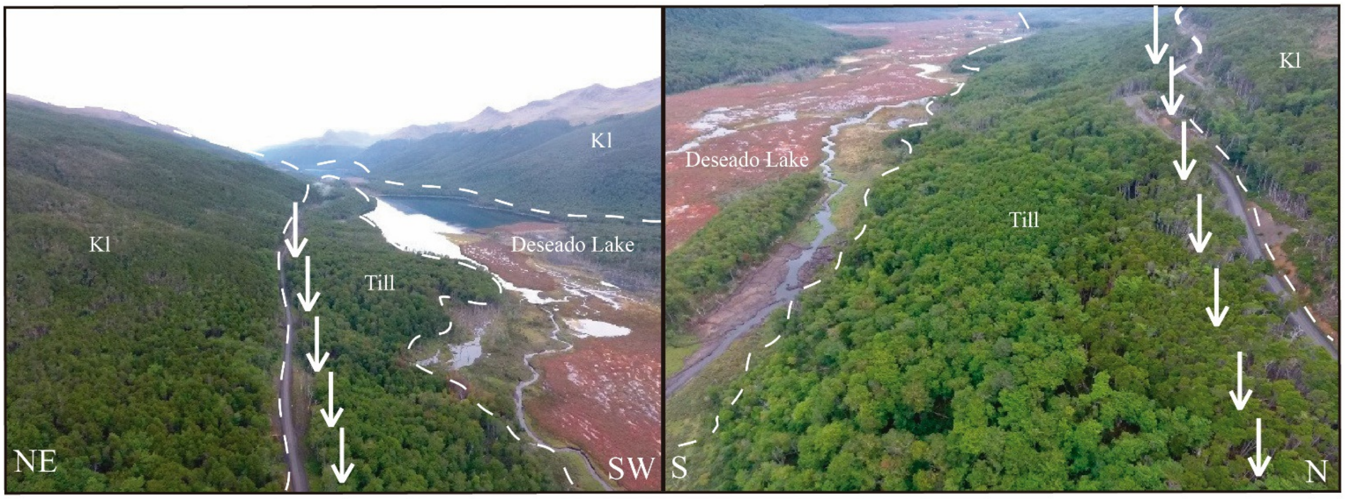

Drone photographs along Deseado fault near the northern slope of Lake Deseado, cutting Upper Cretaceous mudstones of Beavouir Formation and overlying glacial deposits. White arrows show the trace of the fault. Note the change is colour of the vegetation on the right side of the right photo within the till. This is where the DF forms an uphill facing scarp that ponds runoff which creates bogy conditions. Original drone photographs collected by the authors.

Figure S9

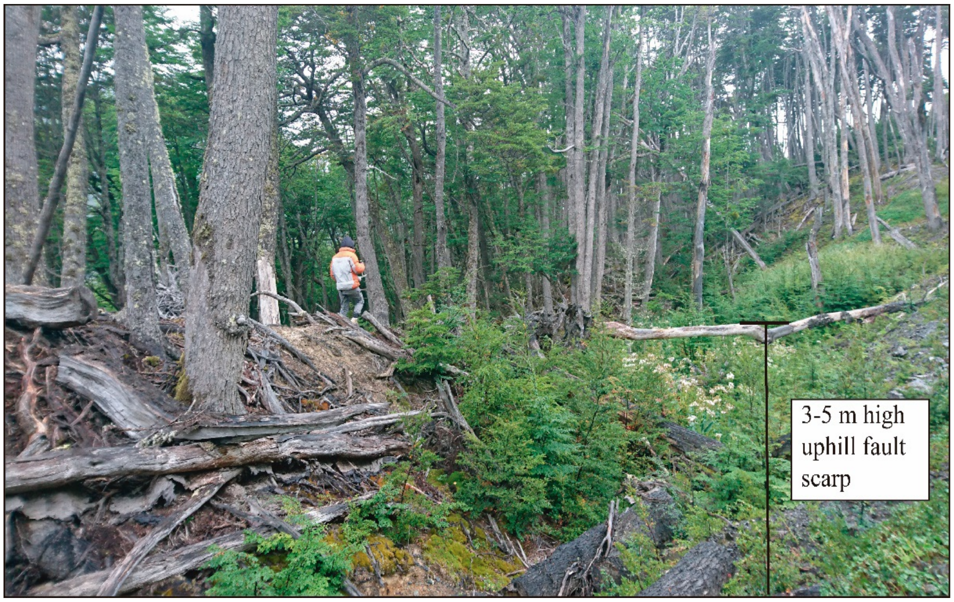

Field photograph showing an uphill facing fault scarp along the Deseado fault (looking NW), along the northern slope of Lake Deseado. Note the trees appear to be tilted along the fault scarp and then are growing vertical (i.e. are deformed from the most recent event tilting their trunks). The clear tectonic geomorphology and due to a recent rupture (post initiation of growth of these trees) here is consistent with a fault with at least a 1 mm/yr slip rate (and perhaps greater). Original field photographs collected by the authors.

Figure S10

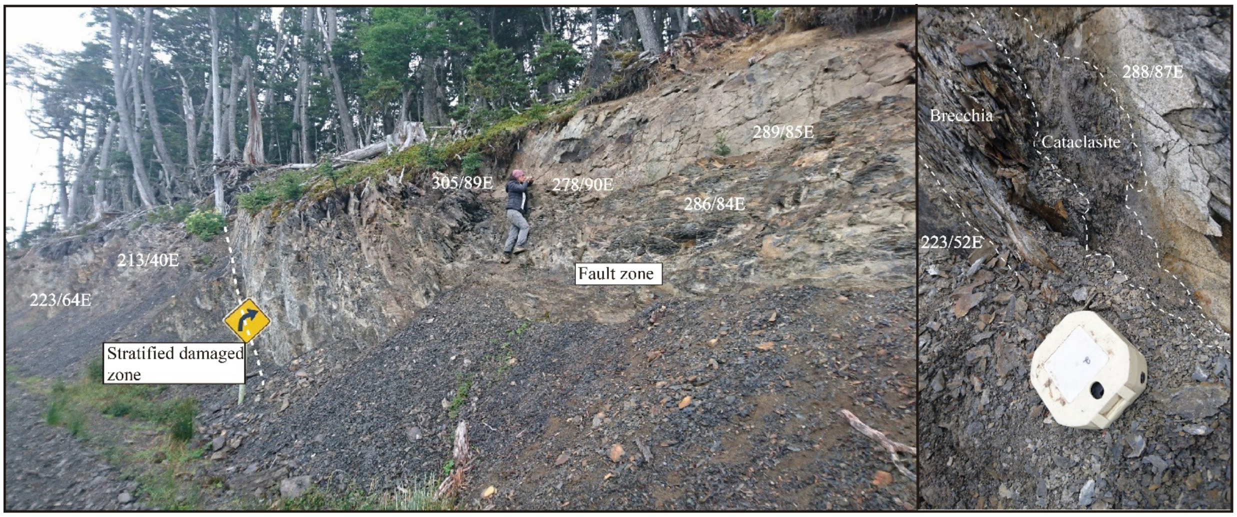

Field photographs of where the Deseado fault cuts sedimentary marine mudstones of the Upper Cretaceous Beauvoir Formation. This site is located within the above airphoto shown in Figure S8 and and the fault in bedrock is coincident with the geomorphic trace shown in Figure S9. Note flat and light brown fault plane to the right of person. Inset shows fault rocks with compass for scale. Original field photographs by the authors.

Figure S11

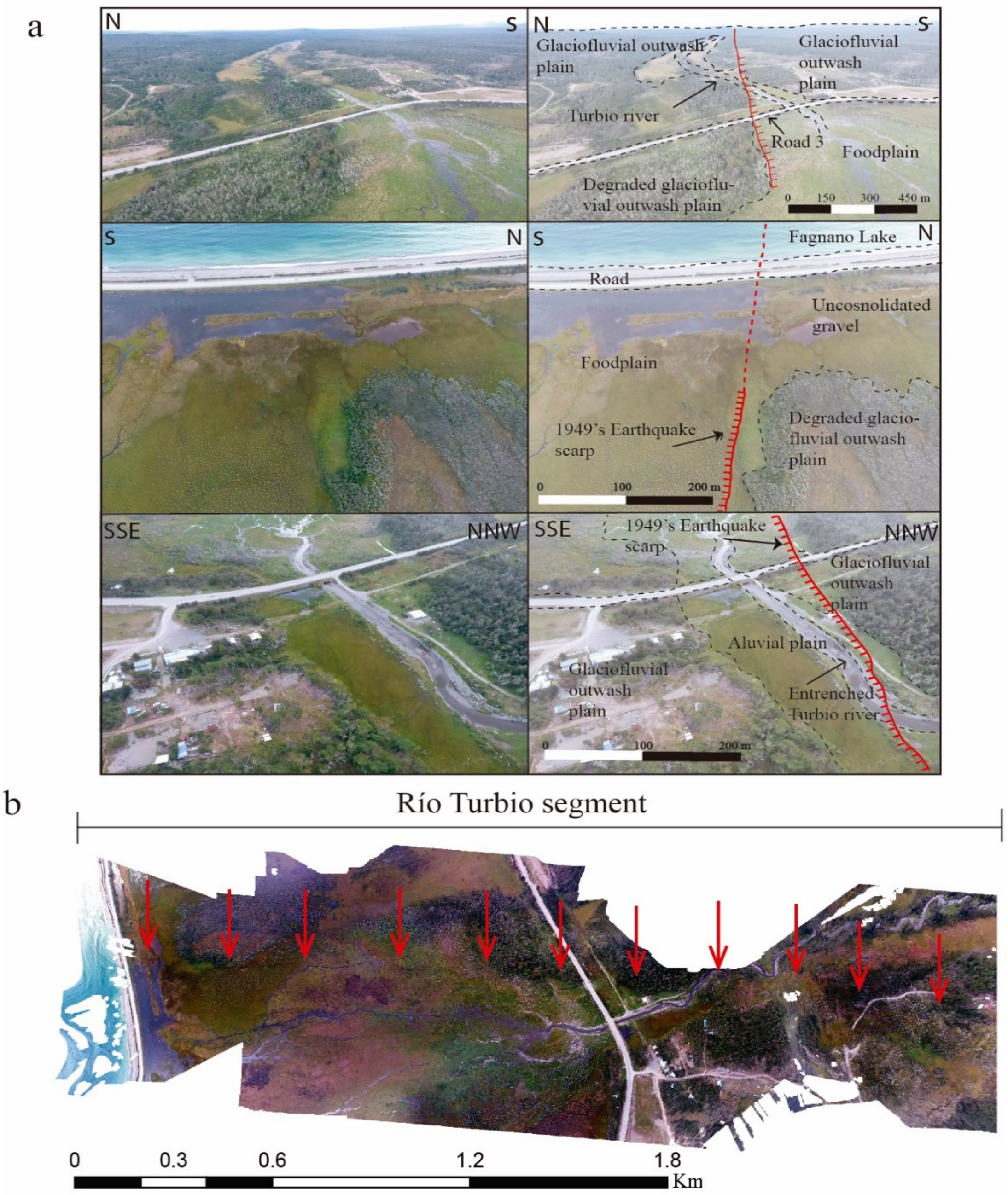

a) Annotated MF Drone photographs from near the eastern shore of Lake Fagnano near Tolhuín, Argentina. b) SfM orthophoto at Río Turbio with the shoreline of Lake Fagnano on right. Red arrows indicates location of the MF surface rupture described by Costa et al., (2006). SfM models were generated using the Agisoft Standard Photoscan Pro 1.3.2 (2018) (https://www.agisoft.com). DEM was processedwith ESRI ArcMap v.10.3 software (under fair terms of use, https://www.esri.com/en-us/legal/copyright-trademarks). All photographs taken by the authors.

Figure S12

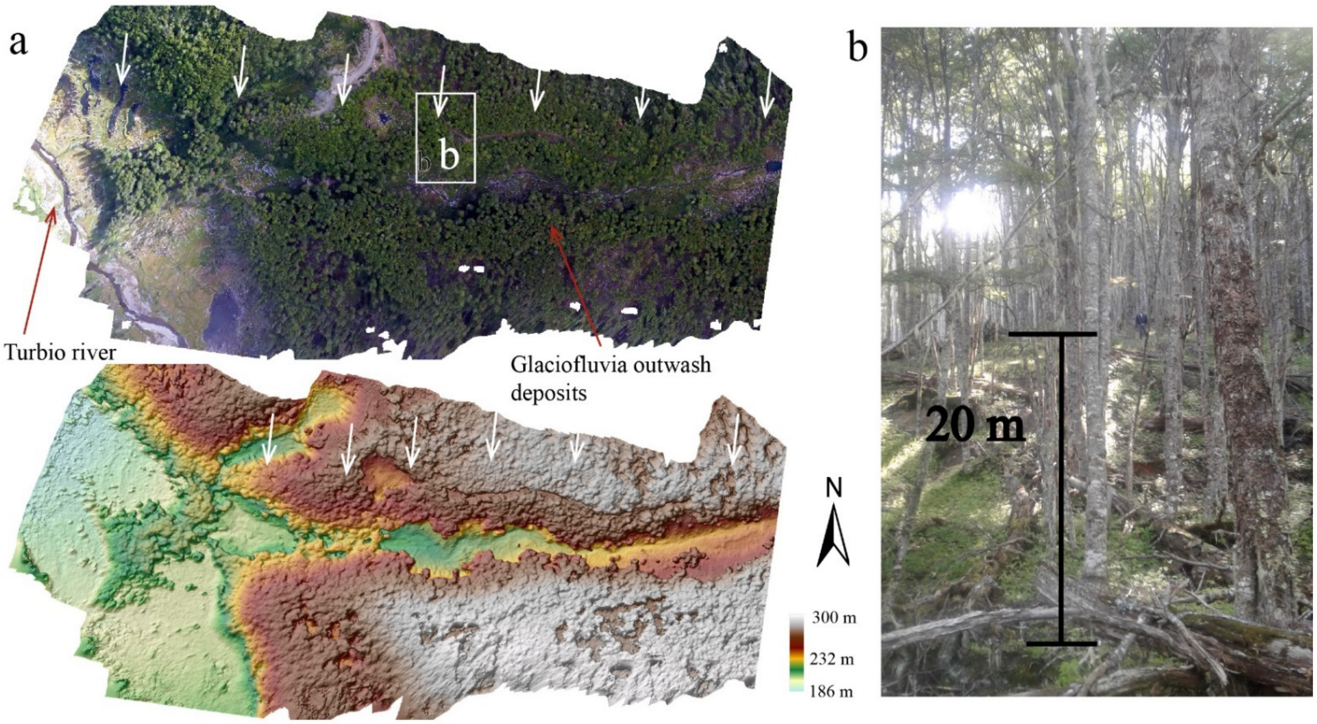

a) Ortophoto and DEM of the eastern portion of Río Turbio valley, Argentina (For location see Figure 4.8), white arrows indicate fault trace location. Note that the fault trace cannot be seen in the DEM nor the othophoto because of the dense forest cover. However in the field, the regional fault trace showed clear tectonic geomorphology with ponded alluvium and shutter ridges. b) 20 meters-high shutter ridge north the fault segment (note tiny person in blue for scale right of the scale bar). SfM models were generated using the Agisoft Standard Photoscan Pro 1.3.2 (2018) (https://www.agisoft.com). DEM was processedwith ESRI ArcMap v.10.3 software (under fair terms of use, https://www.esri.com/en-us/legal/copyright-trademarks).

Table S7: Offset measurement synthesis for the Magallanes Fault in Argentina, in the western arm of Lainez river (These measurements correspond to site in Figure 4).

Argentina-Site 3	Projection 1	Projection 2	Projection 3	Projection 4	Profile
**Confidence degree**	A	A	A	A	B
**Offset (m)**	115±5	118±5	125±5	130±10	110±10
**Geomorphological marker description**	380 m Contour, glaciofluvial outwash deposit	400 m Contour, glaciofluvial outwash deposit	350 m Contour, left limit of Lainez River deposits	350 mContour, right limit of Lainez River deposits	River channel back slip
**Age min = 13.4 ka**					
**Age max = 17.8 ka**					
**Slip-rate for site 3**<br>**7.8±1.3 mm/yr (6.5 to 9.2 mm/yr)**					

Figure S13

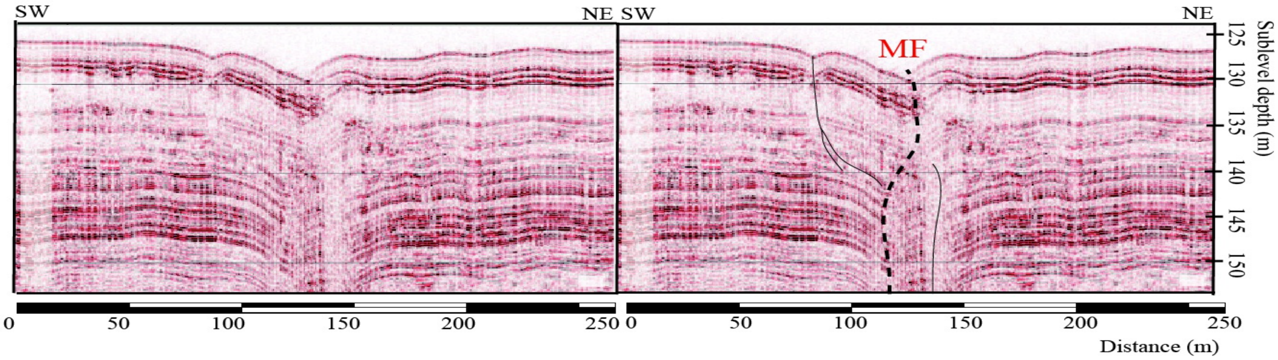

a) NE-SW seismic profile crossing the central sub-basin of Lake Fagnano (after Waldmann et al., 2011). b) Interpreted NE-SW seismic profile crossing the central sub-basin of Lake Fagnano. The black dashed line indicates MF location. For location on floor see light blue star in main paper Figure 8 in the center of the Lake. Modified for this study from data collected and processed from Waldmann et al., (2011).

Table S8: Structure for Motion (SfM) model details

Model	Number of images	Flying altitude (m)	Ground resolution (cm/pix)	Coverage area (km^2)	Camera stations	Tie points	Camera model	Dem resolution (cm/pixel)	Point density (points/m^2)	Focal length (mm)	Pixel size (μm)	XY error (m)	Total error (m)
Caleta María model	213	241	9,2	1,59	213	88,177	FC330	36,8	7,38	3,61	1,56 x 1,56	2,59	3,168
Azopardo valley center model	178	362	14,2	2,2|	178	50,059	FC330	56,7	3,11	3,61	1,56 x 1,56	2,02	4,71
Azopardo valley bridge model	163	288	10,9	1,08	163	59,550	FC330	43,8	5,21	3,61	1,56 x 1,56	2,52	5,612
Hope fault model	202	212	9,3	1,8	212	97,33	FC330	39,4	6,02	3,61	1,56 x 1,56	2,9	5,89
Turbio river segment	313	247	9,45	2,45	310	158,886	FC330	37,8	6,99	3,61	1,56 x 1,56	38	5,118
Lainez river segment	216	265	9,96	1,12	216	148,935	FC330	39,8	6,3	3,61	1,56 x 1,56	2,2	3,47
Turbio river at east model	129	236	0,11	0,53	129	58,8	148,935	36,4	7.54	3,61	1,56 x 1,56	2,53	2,53

Figure S14

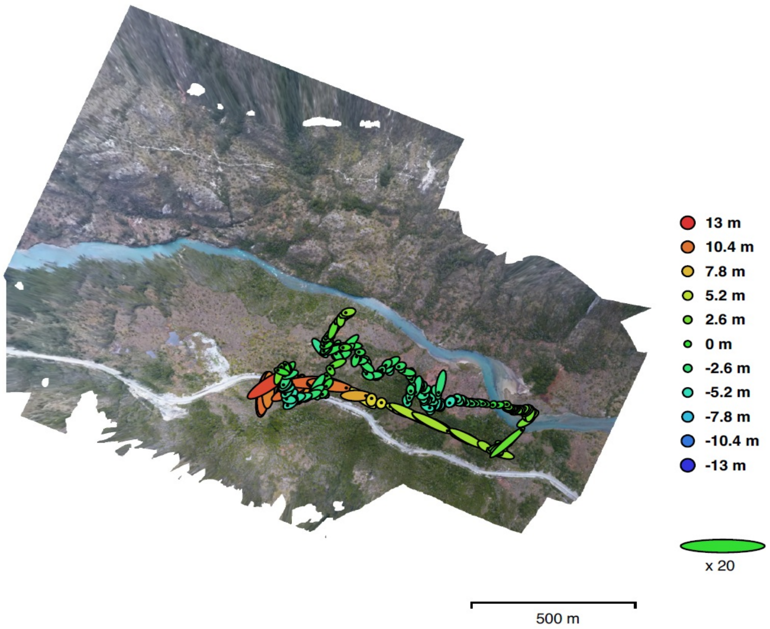

Drone camera locations and error estimates in the central Azopardo Valley. SfM models were generated using the Agisoft Standard Photoscan Pro 1.3.2 (2018) (https://www.agisoft.com).

Figure S15

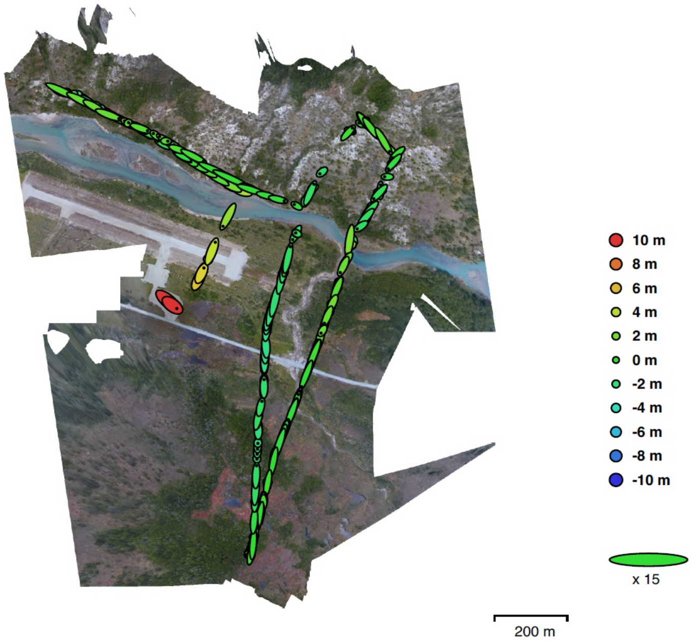

Drone Camera locations and error estimates in in the model near Caleta María, western Azopardo Valley. SfM models were generated using the Agisoft Standard Photoscan Pro 1.3.2 (2018) (https://www.agisoft.com).

Figure S16

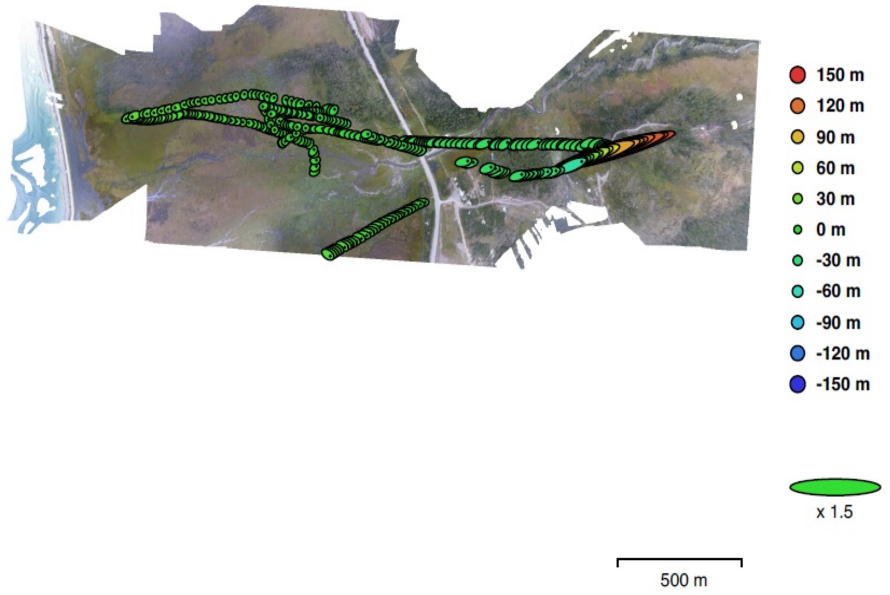

Drone camera locations and error estimates in Rio Turbio Argentina segment model. SfM models were generated using the Agisoft Standard Photoscan Pro 1.3.2 (2018) (https://www.agisoft.com).

Figure S17

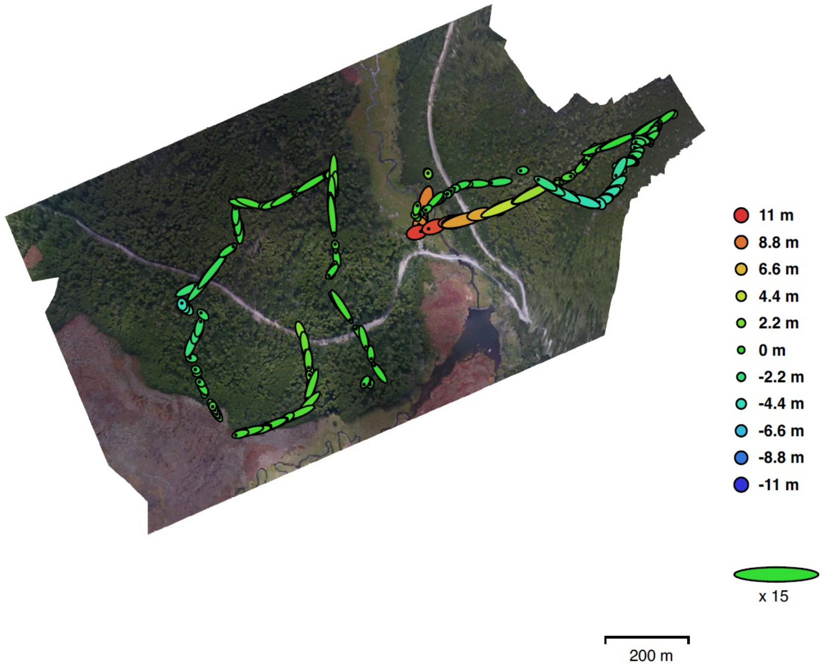

Drone camera locations and error estimates for the Lainez River model in Argentina. SfM models were generated using the Agisoft Standard Photoscan Pro 1.3.2 (2018) (https://www.agisoft.com).

Figure S18

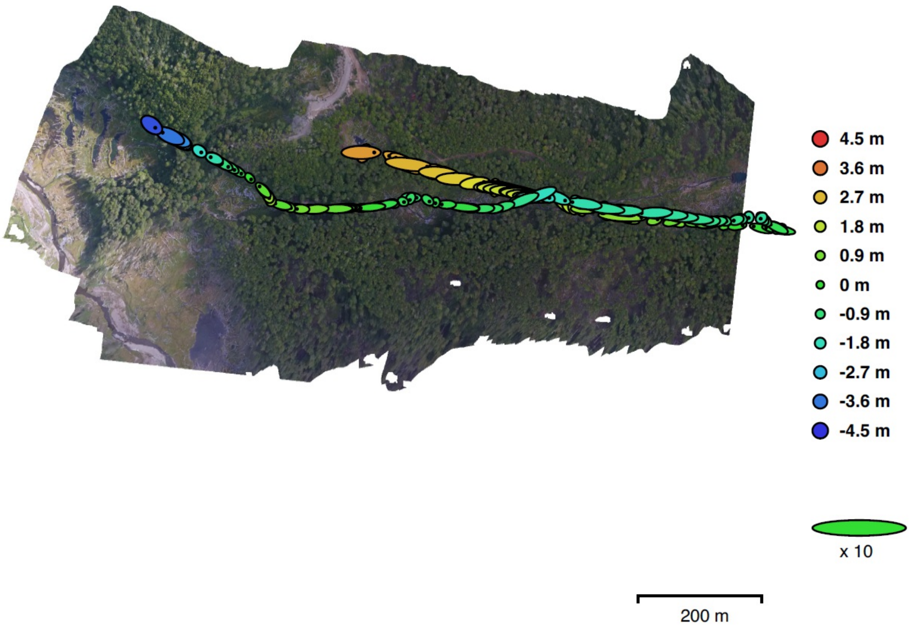

Drone camera locations and error estimates in Eastern Turbio River model in Argentina. SfM models were generated using the Agisoft Standard Photoscan Pro 1.3.2 (2018) (https://www.agisoft.com).

Figure S19

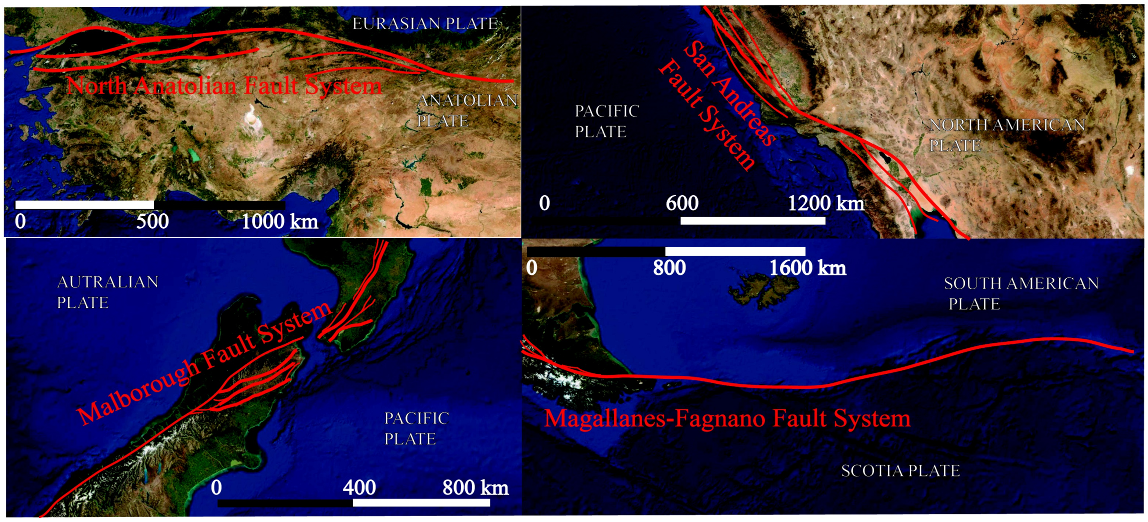

4 major, strike slip plate boundary fault systems. Clockwise from top right, the Northern Anatolian Fault in Turkey, the San Andreas Fault in California, the Magallanes Fault, and the Malborough Fault System in New Zealand. Note that the Magallanes Fault System is apparently the narrowest with crustal plate boundary deformation concentrated along a narrow band of active faults that appear to be < 50 km wide. Further work in Patagonia will evaluate if submarine faults to the south of the MF and HF are active players within the MFS (thus extending the width of deformation). All map data in this figure are from Google, Maxar Technologies (www.google.com/earth).

Supplemental Data files related to the MFS study

These 3D models (i.e. 3D pdf files) of the study sites created using SfM methods (derived from drone photographs) can only be displayed with Adobe Acrobat software as a 3D pdfs. SfM models were generated using the Agisoft Standard Photoscan Pro 1.3.2 (2018) (https://www.agisoft.com).

Data S1: MF in Chile 3D SfM model

This model was used to address the main strand of the MF in Chile along the northwestern shore of Lake Fagnano.

Data S2: Western Azopardo Valley, near Caleta María 3D SfM model

This model was used to search for possible geomorphic offsets along the HF in western Azopardo Valley, Chile.

Data S3: Central Azopardo Valley 3D SfM model

This model was used to measure geomorphic offsets along the HF in the center portion of Azopardo Valley in Chile.

Data S4: Azopardo bridge, eastern Azopardo Valley 3D SfM model

This model was used to measure geomorphic offsets along the HF in the eastern portion of Azopardo Valley, next to Lake Fagnano in Chile.

Data S5: Turbio River Valley in Argentina 3D SfM model

This model was used to search for possible geomorphic offsets along the MF in the Turbio River valley, west Fagnano Lake in Argentina.

Data S6: West Turbio River Valley in Argentina 3D SfM model

This model was used to search for possible geomorphic offsets along the MF west Turbio River valley in Argentina.

Data S7: Lainez River, western arm, in Argentina 3D SfM model

This model was used to search for possible geomorphic offsets along the MF west Turbio River valley in Argentina.

Additional References cited in the *Supplemental Information* (not included in the main paper)

Coronato, A., Meglioli, A., & Rabassa, J. (2005). Glaciations in the Magellan Straits and Tierra del Fuego, Southernmost South America. In Developments in Quaternary Sciences (Vol. 2, pp. 45-48). Elsevier.

Johnson, K., Nissen, E., Saripalli, S., Arrowsmith, J. R., Mcgarey, P., Scharer, K., Williams, P., and Blisniuk, K. 2014. Rapid mapping of ultrafine fault zone topography with structure from motion, Geosphere, 10, 969–986, doi:10.1130/GES01017.1, 2014.

Kurz, C., Thormählen, T., Siedel, H., 2011. Visual Fixation for 3D Video Stabilization. Journal of Virtual Reality and Broadcasting, 8(2):12 p.

Mendoza L., Perdomo R., Hormaechea J.L., Del Cogliano D., Fritsche M., Richter A., Dietrich R. (2011). Present-day crustal deformation along the Magallanes-Fagnano Fault System in Tierra del Fuego from repeated GPS observations. Geophys J Int 184:1009–1022.

Mendoza, L., Richter, A., Fritsche, M., Hormaechea, J. L., Perdomo, R., & Dietrich, R. (2015). Block modeling of crustal deformation in Tierra del Fuego from GNSS velocities. Tectonophysics, 651, 58-65.

Pelayo A., Wiens D. (1989). Seismotectonics and relative plate motions in the Scotia Sea region. J Geophys Res 94:7293–7320.

Rossello E.A. (2005). Kinematics of the Andean sinistral wrenching along the Fagnano-Magallanes Fault Zone (Argentina-Chile Fuegian Foothills). VI International Symposium on Andean Geodynamics, Barcelona, Extended Abstracts, pp 623–626.

Sabbione, N., Connon, G., Hormaechea, J., & Rosa, M. (2007). Estudio de sismicidad en la provincia de Tierra del Fuego, Argentina. Geoacta, 32, 41-50.

Thomas, C., Livermore, R., & Pollitz, F. (2003). Motion of the Scotia Sea plates. Geophysical Journal International, 155(3), 789-804.

Torres-Carbonell, P. J., Olivero, E. B., & Dimieri, L. V. (2008). Control en la magnitud de desplazamiento de rumbo del Sistema Transformante Fagnano, Tierra del Fuego, Argentina. Revista geológica de Chile, 35(1), 63-77.

Waldmann, N., Anselmetti, F. S., Ariztegui, D., Austin Jr, J. A., Pirouz, M., Moy, C. M., & Dunbar, R. (2011). Holocene mass-wasting events in Lago Fagnano, Tierra del Fuego (54 S): implications for paleoseismicity of the Magallanes-Fagnano transform fault. Basin Research, 23(2), 171-190.

Winslow M.A. (1982). The structural evolution of the Magallanes basin and neotectonics in the Southernmost Andes. In: Craddrock C (ed) Antarctic geosciences, symposium on Antarctic geology and geophysics. University of Wisconsin Press, Madison, pp 143–154.
