# Supplementary material for: Slip rates along the narrow Magallanes Fault System, Tierra Del Fuego Region, Patagonia
Source: Sci Rep. 2020 May 18;10:8180. doi: 10.1038/s41598-020-64750-6 (PMC7235261; doi:10.1038/s41598-020-64750-6)
Supplement: Supplementary file 4 — Data S3 - MFS SfM dataset. [file 41598_2020_64750_MOESM4_ESM.pdf]

Copyright GDP - Dept de Geologia - FCFM - UChile
